# Deep learning based high-throughput phenotyping of chalkiness in rice exposed to high night temperature

**DOI:** 10.1186/s13007-022-00839-5

**Published:** 2022-01-22

**Authors:** Chaoxin Wang, Doina Caragea, Nisarga Kodadinne Narayana, Nathan T. Hein, Raju Bheemanahalli, Impa M. Somayanda, S. V. Krishna Jagadish

**Affiliations:** 1 Department of Computer Science, Kansas State University, Manhattan, KS 66506 USA; 2 Institute for Genomics, Biocomputing and Biotechnology, Mississippi State University, Mississippi State, MS 39762 USA; 3 Department of Agronomy, Kansas State University, Manhattan, KS 66506 USA; 4 Department of Plant and Soil Sciences, Mississippi State University, Mississippi State, MS 39762 USA

**Keywords:** Rice grain chalkiness detection, Image segmentation, Convolutional neural networks, Gradient-weighted class activation mapping, High night temperature

## Abstract

**Background:**

Rice is a major staple food crop for more than half the world’s population. As the global population is expected to reach 9.7 billion by 2050, increasing the production of high-quality rice is needed to meet the anticipated increased demand. However, global environmental changes, especially increasing temperatures, can affect grain yield and quality. Heat stress is one of the major causes of an increased proportion of chalkiness in rice, which compromises quality and reduces the market value. Researchers have identified 140 quantitative trait loci linked to chalkiness mapped across 12 chromosomes of the rice genome. However, the available genetic information acquired by employing advances in genetics has not been adequately exploited due to a lack of a reliable, rapid and high-throughput phenotyping tool to capture chalkiness. To derive extensive benefit from the genetic progress achieved, tools that facilitate high-throughput phenotyping of rice chalkiness are needed.

**Results:**

We use a fully automated approach based on convolutional neural networks (CNNs) and Gradient-weighted Class Activation Mapping (Grad-CAM) to detect chalkiness in rice grain images. Specifically, we train a CNN model to distinguish between chalky and non-chalky grains and subsequently use Grad-CAM to identify the area of a grain that is indicative of the chalky class. The area identified by the Grad-CAM approach takes the form of a smooth heatmap that can be used to quantify the degree of chalkiness. Experimental results on both polished and unpolished rice grains using standard instance classification and segmentation metrics have shown that Grad-CAM can accurately identify chalky grains and detect the chalkiness area.

**Conclusions:**

We have successfully demonstrated the application of a Grad-CAM based tool to accurately capture high night temperature induced chalkiness in rice. The models trained will be made publicly available. They are easy-to-use, scalable and can be readily incorporated into ongoing rice breeding programs, without rice researchers requiring computer science or machine learning expertise.

**Supplementary Information:**

The online version contains supplementary material available at 10.1186/s13007-022-00839-5.

## Background

Rice (*Oryza sativa*) is a staple food crop for nearly half the world population [1]. In 2019, the world produced over 750 million tonnes of rice [2], which placed rice as the third highest amongst cereals, only trailing wheat (*Triticum aestivum*) (765 million tonnes) and maize (*Zea mays*) (1.1 billion tonnes). As the global population is expected to reach 9.7 billion by 2050 [3], agricultural production must be doubled in order to meet this demand [4]. As of 2008, rice yields are increasing on average by 1% annually and, at this rate, the production will only increase by 42% by 2050 which falls well short of the desired target [5].

In addition to the required increase in production, climate variability threatens future rice grain yields and quality attributes [6, 7]. Temperatures above 33 °C during anthesis can cause significant spikelet sterility [8–11]. It is predicted that approximately 16% of the global harvested area of rice will be exposed to at least 5 days of elevated temperature during the reproductive period by 2030s [12]. In addition to yield losses, heat stress during the grain-filling period is shown to increase grain chalkiness in rice [13–15]. Disaggregating the mean increase in global temperature has resulted in identifying a more rapid increase in the average minimum night temperature than the average maximum day temperature [16]. High night temperature stress during the grain-filling period can lead to severe yield and quality penalties, primarily driven by increased night respiration [17–19]. An increased rate of night respiration during grain-filling ultimately impairs grain yield and quality through reduction in 1000 grain weight, grain width, reduced sink strength with lowered sucrose and starch synthase activity resulting in reduced grain starch content, and an increase in rice chalkiness [13, 19–21].

“Chalkiness is the opaque part of the milled rice grain and is one of the key factors that determines rice grain quality^1^.” More specifically, chalkiness is the visual appearance of loosely packed starch granules [13, 22]. The poor packaging of starch granules leads to an increased number of oversized air pockets within the grain. The air pockets prevent reflection, giving the chalky portions of the grains an opaque appearance [23]. Chalkiness is an undesirable trait, and an increased proportion of chalk leads to a linear decrease in the market value of rice [15]. In addition, high levels of chalk lead to increased breakage during milling and degrade cooking properties, and lower palatability [14, 15, 22, 24].

Three different processes have been considered to explain the cause of increased chalkiness under heat stress: (1) a reduction in carbon capture, photosynthetic efficiency, or the duration of the grain-filling period inhibits the plant’s ability to provide a sufficient amount of assimilates to the seed, (2) reduced activity of starch metabolism enzymes, which are used to convert sugars to starch, and (3) hormonal imbalance between ABA and ethylene as a high ABA-to-ethylene ratio is vital during grain-filling [25]. Physiologically, the level of chalkiness is dependent on the source-sink relationships, with the primary tillers in rice having greater advantage of accessing the carbon pool compared to later formed tillers. We tested the hypothesis that, under higher night temperatures, increased carbon loss due to higher respiration would lead to different levels of grain chalkiness among the tillers with the least chalkiness from primary panicles and the highest chalkiness in the later formed tillers. Regardless of the cause or differential chalkiness among tillers, the ability to quickly and accurately identify and quantify the chalkiness in rice is extremely important to help not only to understand the cause of chalkiness, but also to breed for heat tolerant nutritional rice varieties [19, 26–28].

Traditional grain phenotyping has been performed by manual inspection [29]. As such, it is subjective, inefficient, tedious, and error-prone despite the fact that it is performed by a highly skilled workforce [30]. Over the past decade, interest has grown in applying image-based phenotyping to provide quantitative measurements of plant-environment interactions with a higher accuracy and lower labor-cost than previously possible [31].

In particular, several automated approaches for rice grain chalkiness classification, segmentation and/or quantification have been developed. For example, the K-means clustering approach performs instance segmentation (i.e., identifies the pixels that belong to each instance of an object of interest, in our case “chalkiness”) by grouping pixels based on their values [32]. One advantage of the K-means clustering approach is that it works in an unsupervised manner and does not require manually labeled ground truth [33]. However, one disadvantage is that it involves extensive parameter tuning to identify good clusters corresponding to objects of interest in an image. Furthermore, the final clusters depend on the initial centroids and the algorithm needs to be run several times with different initial centroids to achieve good results [34].

In addition to the K-means clustering approach, threshold based approaches have been used for chalkiness identification and quantification. For example, a multi-threshold approach based on maximum entropy was used for chalky area calculation [35] and another threshold-based approach was used to detect broken, chalky and spotted rice grains [36]. However, such approaches need extensive fine-tuning to identify the right thresholds and are not easily transferable to seeds of different types or to images taken under different conditions. Support vector machine (SVM) approaches have been used to classify grains according to the location of the chalkiness [37], and to estimate rice quality by detecting broken, chalky, damaged and spotted grains in red rice based on infrared images [38]. Similar to the threshold-based approaches, the SVM classifiers are not easily transferable to images containing different types of seeds or taken under different illumination conditions. Furthermore, they require informative image features to be identified and provided as inputs to produce accurate results. Rice chalkiness has also been addressed using specially designed imaging instruments. For example, Armstrong et al. used a single–kernel near–infrared (SKNIR) tube instrument and a silicon–based light–emitting diode (SiLED) high–speed sorter to classify single rice grains based on the percentage of chalkiness [39]. Unfortunately, the single-kernel approach is limited in scope and cannot be used to develop a high-throughput phenotyping method. More recently, volume based quantification technologies, such as X-ray microcomputed tomography, have been used to quantify rice chalkiness [27]. However, such technologies are extremely expensive and, thus, are beyond the reach of routine crop improvement programs and for traders and millers who regularly estimate chalkiness and establish a fair market price.

In recent years, the use of deep learning approaches for image classification and segmentation crop science tasks have led to state-of-the-art high-throughput tools that outperform the results from traditional machine learning and image analysis techniques [40, 41], enabling researchers to capture a wide range of genetic diversity [42]. To the best of our knowledge, deep learning approaches have not been used to detect chalkiness despite being used to address other challenging problems in crop science. To address this need, we investigated modern deep learning techniques to create a tool that facilitates high-throughput phenotyping of rice chalkiness to support genetic mapping studies and enable development of rice varieties with minimal chalkiness under current and future warming scenarios. One possible solution to rapidly and accurately phenotype chalkiness is provided by Mask R-CNN [43]. Mask R-CNN is a widely used instance detection and segmentation approach, which employs a convolutional neural network (CNN) as its backbone architecture. One limitation of the Mask R-CNN approach is that it requires pixel-level ground truth with respect to the concept of interest, in our case, chalkiness. Acquiring pixel-level ground truth is laborious and expensive [44]. Furthermore, the Mask R-CNN segmentation approach labels the pixels of a rice grain as chalky or non-chalky, while sometimes it may be preferable to characterize the pixels based on the chalkiness intensity, i.e., on a continuous scale as opposed to a binary scale.

To address the limitations of the Mask R-CNN approach, we framed the problem of detecting chalkiness as a binary classification problem (i.e., a grain is chalky or non-chalky) and used CNNs combined with class activation mapping, specifically Grad-CAM [45], to identify the chalkiness area in an image. Grad-CAM works on top of a CNN model for image classification. It makes use of the gradients of a target category to produce a heatmap that identifies the discriminative regions for the target category (i.e., regions that explain the CNN model prediction) and implicitly localizes the category in the input image. By framing the problem as an image classification task, Grad-CAM can help reduce the laborious pixel-level labeling task to a relatively simpler image labeling task, i.e., an image is labeled as chalky or non-chalky. Furthermore, the heatmaps produced by Grad-CAM have soft boundaries showing different degrees of chalkiness intensity. The values of the pixels in a heatmap can be used to calculate a chalkiness intensity score corresponding to an image. This weakly supervised approach to segmentation was originally proposed by Oquab et al. [46] and has been used in other application domains [47–51], including in the agricultural domain for segmentation of citrus pests [52] and for remote sensing imagery [53], among others. Such approaches are generally called weakly supervised semantic segmentation approaches, given that they only require image-level labels as opposed to pixel-level labels.

The Grad-CAM based approach to rice chalkiness detection has the potential to help rice phenomics catch up with the developments in rice genomics [54] as well as help implementing new advances in achieving the target of nutritious food production goals by 2050 [55]. To summarize, the contributions of this research are:

- We proposed to use a weakly supervised approach, Grad-CAM, to classify rice grains as chalky or non-chalky and subsequently detect the chalkiness area in chalky grains.
- We experimented with the Grad-CAM approach (with a variety of CNN networks as backbone) on polished rice seeds and evaluated the performance using both instance classification and segmentation metrics as well as time and memory requirements.
- We compared the weakly supervised Grad-CAM approach with the Mask R-CNN segmentation approach on polished seeds and studied its transferability to unpolished rice seeds (i.e., to rice seeds that have not been polished after the removal of the husk).
- We tested the applicability of the tool in determining the level of chalkiness in rice plants exposed to high night temperature (HNT) and quantified the differential level of chalkiness among tillers within a plant exposed to HNT stress.

## Methods and materials

### Deep learning methods for rice chalkiness segmentation

We address the rice chalkiness segmentation problem using a weakly supervised Grad-CAM approach, which requires binary (chalky or non-chalky) image-level labels as opposed to more expensive pixel-level labels.

#### Overview of the approach

**Fig. 1:**
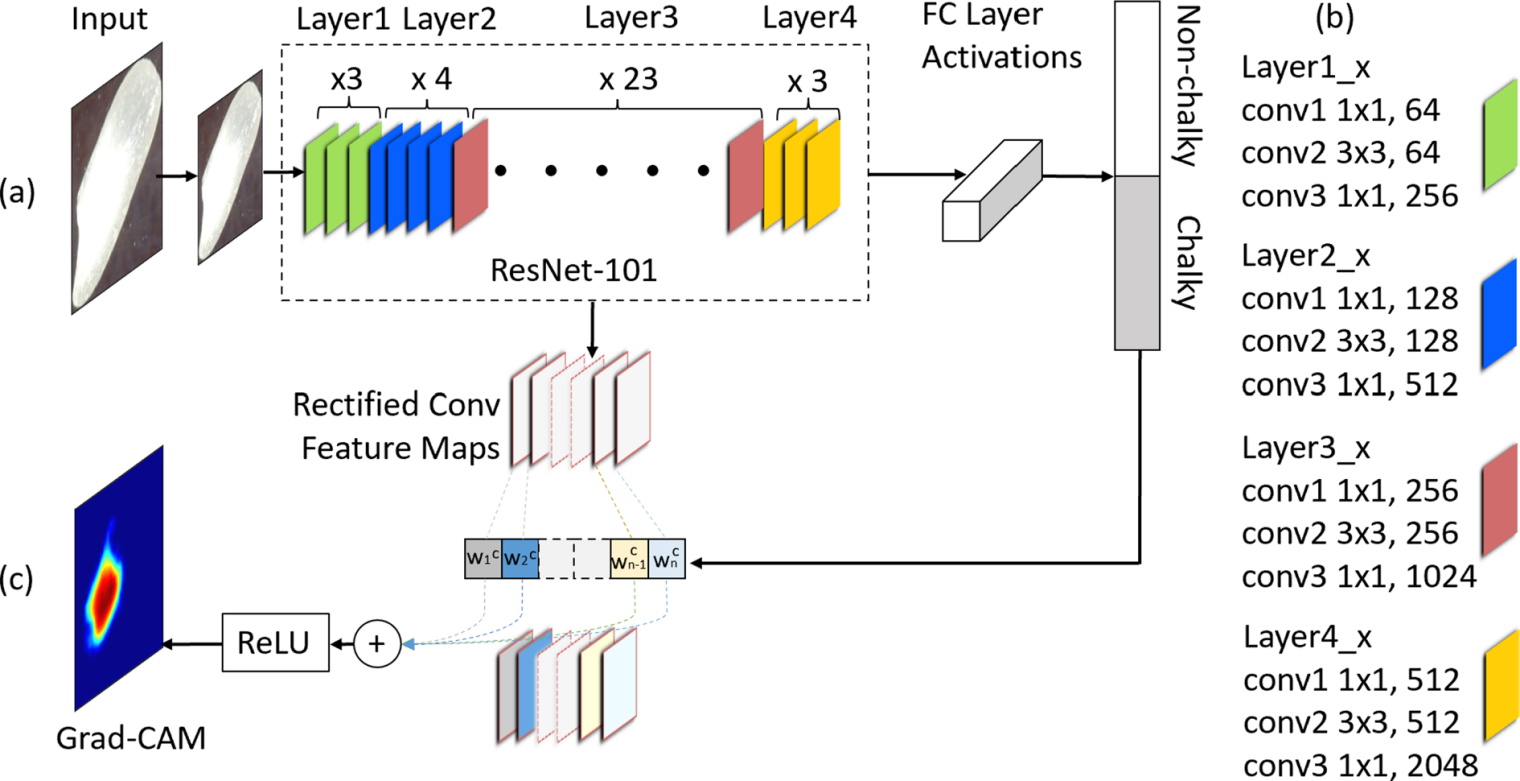
Model Architecture. **a** A backbone CNN (e.g., ResNet-101) is trained to classify (resized) input grain images as chalky or non-chalky. ResNet-101 has four main groups of convolution layers, shown as Layer1, Layer2, Layer3, and Layer4, consisting of 3, 4, 23 and 3 bottleneck blocks, respectively. **b** Each bottleneck block starts and ends with a $1\times 1$ convolution layer, and has a $3\times 3$ layer in the middle. The number of filters in each layer is shown after the kernel dimension. **c** Grad-CAM uses the gradients of the chalky category to compute a weight for each feature map in a convolution layer. The weighted average of the features maps, transformed using the ReLU activation, is used as the heatmap for the current image at inference time

The Grad-CAM approach includes two main components: (i) a deep CNN network (e.g., VGG or ResNet) that is trained to classify seed images into two classes, chalky or non-chalky; and (ii) a class activation mapping component, which generates a rice chalkiness heatmap as a weighted average of the feature maps corresponding to a specific layer in the CNN network. The chalkiness heatmap can be further used to calculate a chalkiness score, which quantifies the degree of chalkiness in each individual grain, and to estimate the chalkiness area for each grain. An overview of the approach is shown in Fig. 1. Details for the components of the model are provided below.

#### CNNs

Models based on CNNs have been successfully used for many image classification and segmentation tasks [56–58]. A CNN consists of convolutional layers (which apply filters to produce feature maps), followed by non-linear activations (such as Rectified Linear Unit, or ReLU), pooling layers (used to reduce the dimensionality), and fully connected layers (that capture non-linear dependencies between features). The last fully connected layer in a classification network generally uses a softmax activation function and has as many output neurons as the number of target classes (in our case, two classes: chalky and non-chalky).

The ImageNet competition (where a dataset with 1.2 million images in 1000 categories was provided to participants) has led to many popular architectures, including highly competitive architectures in terms of performance as well as cost-effective architectures designed to be run efficiently on low-cost platforms generally present in embedded systems [59]. We anticipate that our rice chalkiness detection models could be useful in both environments with rich computational resources and also environments with more limited resources. Thus, given the trade-off between model performance (i.e., accuracy) and model complexity (e.g., number of parameters, memory and time requirements), we consider a variety of networks (and variants) published between 2012 and 2019 including AlexNet [60], Very Deep Convolutional Networks (VGG) [61], Deep Residual Networks (ResNet) [62], SqueezeNet [63], Densely Connected Convolutional Networks (DenseNet) [64], and EfficientNet [65].

#### Grad-CAM approach

The Grad-CAM approach was originally proposed by Selvaraju et al. [66] in the context of understanding the predictions of a CNN model. In recent years, this approach and its variants have been frequently used for weakly supervised object localization [67]. Given a trained CNN model and an input image at inference time, the Grad-CAM approach uses the gradients of a category of interest (specifically, the corresponding logit provided as input to the softmax function) to compute a category-specific weight for each feature map in a selected convolution layer. Formally, let $f^k$ (with $k = 1,\ldots , K$) be a feature map in a particular convolutional layer, which consists of a total of *K* feature maps. Let $y^c$ be the logit (i.e., input to the softmax function) of the class of interest, *c* (e.g., chalky). Grad-CAM averages the gradients of $y^c$ with respect to all *N* pixels $f^k_{ij}$ of the features map $f^k$ to calculate a weight $w^c_k$ representing the importance of the feature map $f^k$. Specifically, $\displaystyle w^c_k = \frac{1}{N} \sum _{i,j} \frac{\partial y^c}{\partial f^k_{i,j}}$. The feature maps $f^k$ of the selected convolutional layer are averaged into one final heatmap for the category of interest, *c*, according to the importance weights $w^k_c$, i.e., $\displaystyle H^c={F} \left( \sum _k w^k_c f^k\right)$, where *F* is a non-linear activation function. Traditionally, ReLU has been used as the activation function to cancel the effect of the negative values while emphasizing areas that positively contribute to the category *c*. The heatmap, $H^c$, is resized to the original input size using linear interpolation. The resized heatmap, $H^c_{final}$, can be used to identify the discriminative regions for the category of interest, *c*, and implicitly to localize the category in the input image. More specifically, the localization is obtained by binarizing the input image using a threshold *T* on the values of the pixels in the heatmap as first proposed by Zhou et al. [68]. The value of the threshold *T* is dependent on the data and task at hand, but can be found using a trial-and-error process as shown in related prior works [69–72]. Regarding the convolutional layer used to produce the heatmao, the last layer was originally used by Selvaraju et al. [66], under the assumption that the last layer captures the best trade-off between high-level semantic features and spatial information. However, in this study, we experimented with a variety of convolutional layers, from lower level convolutional layers (more general) to higher level convolutional layers (more specific), to identify sets of features maps that best capture chalkiness.

#### Variants of the Grad-CAM approach

Grad-CAM is a strong candidate for being used as an explainability/localization approach in the weakly supervised framework, as evidenced by many recent studies that have employed Grad-CAM [48, 52, 73–76]. However, several extensions and variants of the Grad-CAM approach have been proposed, e.g. Grad-CAM++ [77] and Score-CAM [78]. Grad-CAM++ is a direct extension of Grad-CAM which was designed to address two limitations: (1) the fact that Grad-CAM does not properly identify/localize all occurrences of a class object; and (2) the fact that Grad-CAM may not always localize the whole object instance, but only parts of it. Score-CAM is an alternative class activation mapping approach which aims to get away with the use of gradients, as they can be noisy or may vanish when dealing with deep neural networks. Instead of using gradients to weight activation maps, Score-CAM proposes to use weights that correspond to the forward pass scores of the original images perturbed with upsampled activation maps on the target class. We compared Grad-CAM with the Grad-CAM++ and Score-CAM variants to understand if a newer approach can be used to further improve the results obtained using Grad-CAM.

#### Application of Grad-CAM to rice chalkiness detection

 We used the Grad-CAM approach to generate chalkiness heatmaps for rice grain images. The heatmaps show the concept of chalkiness using soft boundaries through a color gradient. This representation is very appropriate for localizing the concept of chalkiness, which exhibits different levels of intensity and, thus, has inherently soft boundaries that separate the chalky area from the non-chalky area. The heatmap, $H^{chalky}_{final}$, corresponding to a particular convolutional layer (determined using validation data) is the final rice chalkiness heatmap and can be used to visualize the area of a seed that is discriminative with respect to chalkiness. This heatmap can further be converted into a chalkiness score corresponding to a rice grain as follows: $ChalkyScore = \frac{1}{Z} \sum _{i}\sum _{j}(H^{chalky}_{final}\cap GrainArea)$, where *Z* represents the total number of pixels in the *GrainArea* in the original image. The resulting chalkiness score has a numerical value between 0 and 1, where 0 means that the grain shows no chalkiness and 1 means that the grain has severe chalkiness all over its surface. Finally, the heatmap is used to create a binary mask for the chalkiness area using a threshold on the intensity of the pixels (determined based on validation data). The masked area can be used to estimate the area of the chalkiness as a percentage of the total grain area. The numeric scores, including the chalkiness score and the chalkiness area, obtained from large mapping populations can be used in determining the genetic control of chalkiness in rice.

#### Baseline approach—mask R-CNN

Mask R-CNN is an object instance segmentation approach, i.e., an approach that identifies instances of given objects in an image (in our case, the chalkiness concept) and labels their pixels accordingly. Mask R-CNN extends an object detection approach, specifically Faster R-CNN [79], to perform instance segmentation. The Faster R-CNN network first identifies Regions of Interest (ROI, i.e., regions that may contain objects of interest) and their locations (represented as bounding box coordinates) using a Region Proposal Network (RPN). Subsequently, the Faster R-CNN network classifies the identified regions (corresponding to objects) into different classes (e.g., chalkiness and background) and also refines the location parameters to generate an accurate bounding box for each detected object. In addition to the object classification and the bounding box regression components of the Faster R-CNN, the Mask R-CNN network has a component for predicting instance masks for ROIs (i.e., identifying all pixels that belong to an object of interest). One advantage of the Mask R-CNN approach is that it is specifically trained to perform instance segmentation and, thus, produces a precise mask for objects of interest. The main disadvantage of the Mask R-CNN baseline, as compared to the weakly supervised Grad-CAM approach, is that it requires expensive pixel-level annotation for training. We compared the weakly supervised Grad-CAM approach to chalkiness segmentation with Mask R-CNN in terms of performance and also time and memory requirements. We have selected the Mask R-CNN approach as a strong baseline for the weakly supervised Grad-CAM approach, given that Mask R-CNN has been shown to be a very competitive approach for instance segmentation in many application domains [80–85].

### High night temperature stress experiment

In this section, we describe plant materials and the biological experiment that generated the data (i.e., rice grains) used in this study.

#### Plant materials

Six genotypes (CO-39, IR-22, IR1561, Oryzica, WAS-174, and Kati) with contrasting chlorophyll index responses to a 14-day drought stress initiated at the agronomic panicle-initiation stage were used in this study [86]. The experiment was carried out in controlled environment chambers (Conviron Model CMP 3244, Winnipeg, MB) at the Department of Agronomy, Kansas State University, Manhattan, KS, USA.

#### Crop husbandry and high night temperature stress imposition

Seeds obtained from the Germplasm Resources Information Network (GRIN) database were sown at a depth of 2 cm in pots (1.6-L, 24 cm tall and 10 cm diameter at the top, MT49 Mini-Treepot) filled with farm soil. Seedlings were thinned to one per pot at the three-leaf stage. Controlled-release Osmocote (Scotts, Marysville, OH, USA) fertilizer (19% N, 6% P_2_O_5_, and 12%K_2_O) was applied (5 g per pot) before sowing along with 0.5 g of Scotts Micromax micronutrient (Hummert International, Topeka, KS) at the three-leaf stage. The plants were well-watered throughout the experiment and a 1–cm water layer was maintained in the trays holding the pots. Seventy-two plants were grown with at least 12 plants per genotype wherein 6 plants were used for control and the remainder for HNT. Plants were grown in controlled environment chambers maintained at control temperatures of 30/21 °C (maximum day/minimum night temperatures; actual inside the chamber: 32.6 °C [SD±1.0]/21.1 °C [SD±0.3]) and relative humidity (RH) of 70% until treatment imposition. Both control and HNT chambers were maintained at a photoperiod of 11/13 h (light/dark; lights were turned on from 0700 to 1800 h, with a dark period from 1800 to 0700 h) with a light intensity of  850 ${\mu }mol$ $m^{-2}$ $s^{-1}$ above the crop canopy. Temperature and RH were recorded every 15 min using HOBO UX 100-011 temperature/RH data loggers (Onset Computer Corp., Bourne, Massachusetts) in all growth chambers. At the onset of the first spikelet opening, the main tiller, primary tillers and other tillers of the flowering genotype were tagged and readied for treatment imposition. The same approach was followed for all six genotypes and replicates. Tagged replicate plants were moved to HNT (30/28 °C) chambers and equal numbers of plants were similarly tagged and maintained in control conditions. Six independent plants for each genotype were subjected to HNT stress (30/28 °C- day/night temperatures; actual: 31.8 °C [SD±0.8]/27.9 °C [SD±0.1]) after initiation of flowering on the main tiller until maturity to determine the impact of HNT on chalkiness while the other six plants were maintained under control conditions.

#### Data collection

At physiological maturity, the plants were harvested from both the control and HNT treatments. The panicles were separated into main panicles (the panicle on the main tiller), two primary panicles (tillers that followed the main panicle), and other remaining panicles for each plant from each treatment and hand threshed separately. Subsequently, the grains were de-husked using the Kett, Automatic Rice Husker TR-250.

In addition to the unpolished grains, polished grains were also used in the initial model development and testing, as polished grains are easier to analyze and label with respect to chalkiness and could potentially be beneficial in terms of knowledge transfer to unpolished rice. The polished grains were obtained from Rice Research and Extension Center in Stuttgart Arkansas, University of Arkansas for preliminary testing and to establish the model. The polished rice grains composed of both medium and long grain rice. For each grain size, there are three degrees of grain chalkiness (roughly estimated by a domain expert): low, medium, and high chalkiness. Thus, based on grain size and degree of chalkiness, the grains were grouped into six categories: (1) long grain, low chalkiness; (2) long grain, medium chalkiness; (3) long grain, high chalkiness; (4) medium grain, low chalkiness; (5) medium grain, medium chalkiness; and (6) medium grain, high chalkiness.

### Rice grain image acquisition and processing

#### Image acquisition

Both polished and unpolished grain samples were arranged in transparent 90 mm Petri-plates with three Petri-plates for each sample. A sample corresponds to a size/chalkiness combination in the case of polished rice and a genotype/tiller/condition combination in the case of unpolished rice. Three replicates (i.e., sets of grains to be used in one scan) were randomly selected (without replacement) for each sample. The grains were scanned using an Epson Perfection V800 photo scanner attached to a computer (see Additional file 1: Fig. S1). Images were scanned at a resolution of 800 dots per inch (dpi) and saved in the TIFF (.tif) file format for further image analysis. A total of 18 (i.e., $3 \times 2 \times 3$) images were acquired for polished rice, and 108 (i.e., $3\times 6\times 3\times 2$) images for unpolished rice. The scanned images included all borders of the three Petri-plates but not excessive blank area outside of the dishes, as shown in Additional file 2: Fig. S2.

#### Image preprocessing

**Fig. 2:**
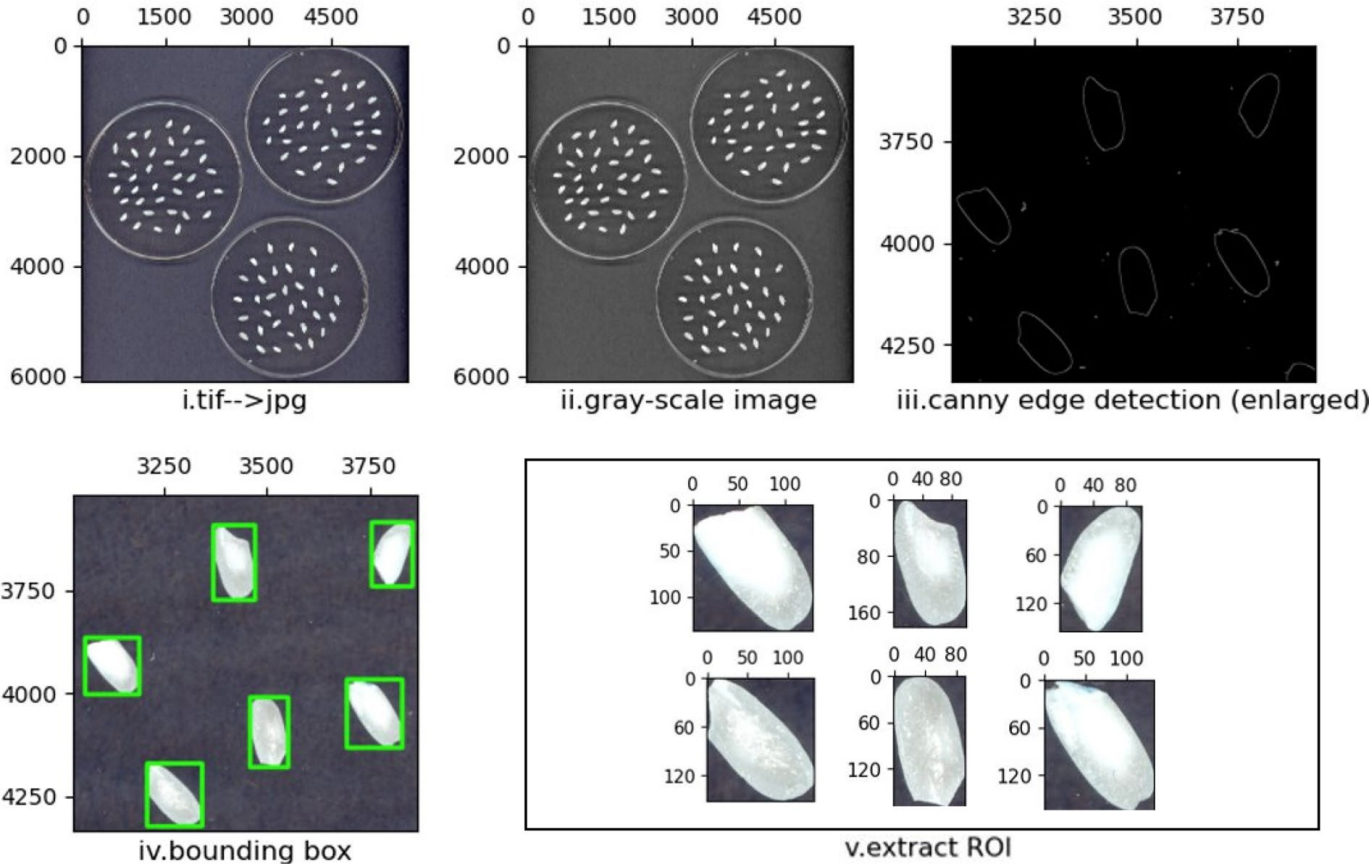
Image preprocessing. Steps used to crop individual rice seeds from the original scanned images, each with approximately 25–30 seeds. Five steps (i. to v.) are depicted below each image that illustrate the action achieved in each respective step

Each scanned image (for both polished and unpolished rice grains) was approximately $6000\times 6000$ pixels. This size is extremely large for deep learning approaches, which require GPU acceleration [87]. Furthermore, as we aim to perform chalkiness detection at grain level using a weakly supervised approach, we need images that contain individual seeds. To reduce the size of the images and to enable grain level labeling and analysis, we resorted to cropping individual grains from the original Petri-plate images (which contain approximately 25–30 rice grains per plate). The following steps, illustrated in Fig. 2, were used to crop individual grain images: (i) we first converted original images from .tif to .jpg format; (ii) converted RGB images to grayscale images; (iii) performed canny edge detection; (iv) identified bounding boxes corresponding to individual seeds; (v) extracted ROIs defined by the bounding boxes and saved each ROI/grain as an image into a file with unique file name.

The total number of individual seeds extracted from the images containing Petri-plates with polished rice grains was 1645 out of the total of 1654 grains in the original set of 18 images. Nine seeds got truncated and were removed from the dataset. The exact number of polished seeds in each image and the corresponding number of extracted seed images are shown in Additional file 3: Table S1 in columns 4 (Grains original) and 5 (Grains used). Similarly, the total number of individual seeds extracted from the images containing Petri-plates with unpolished rice grains was 13,101 out of the total of 13,149 seeds in the original set of 108 high resolution images. In this case, 48 seeds got truncated and were not included in the final set. The exact number of unpolished seeds in each of the 108 images and the corresponding number of individual seed images extracted are shown in Additional file 4: Table S2 in columns 5 (Grains original) and 6 (Grains used).

### Image annotation and benchmark datasets

#### Ground truth labeling

**Fig. 3:**
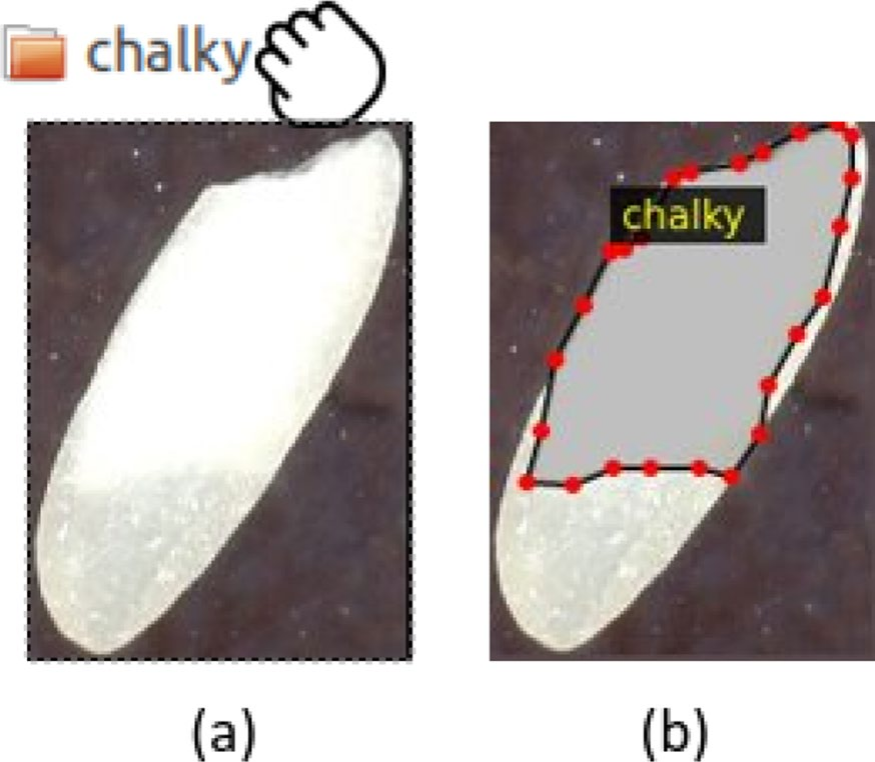
Manual annotations. **a** Image-level annotation: each seed is labeled as chalky or non-chalky (technically, the label was created by dragging each rice seed image into chalky or non-chalky folder, respectively). **b** Specific chalkiness annotation: chalkiness area is marked with polygons using VGG Image Annotator (each red dot in the image represents a click). The dark white opaque region in panel “a” is the chalk portion while the non-chalky region is translucent

Two types of manual annotations were performed and used as ground truth in our study, as shown in Fig. 3. First, for the Grad-CAM weakly supervised approach to chalkiness segmentation, we labeled each rice grain image as chalky or non-chalky. The labeling was done based on visual inspection of the images by a domain expert. Second, to train Mask R-CNN models, which inherently perform instance segmentation, and to evaluate the ability of the Grad-CAM approach to accurately detect the chalkiness area in a rice grain, we manually marked the chalkiness area using polygons. The polygon annotation was performed by a domain expert using the VGG Image Annotator [88], a web-based manual annotation software. Compared to the image-level labeling (i.e., chalky/non-chalky), the polygon annotation is significantly more expensive, as it requires 10 to 100 clicks to draw the polygons, given the irregular shape of the chalkiness area.

Out of 1645 polished grains used in our study, 660 grains were labeled as chalky and 985 grains were labeled as non-chalky. The exact numbers of chalky and non-chalky grains in each of the eighteen high-resolution images with polished rice are shown in Additional file 3: Table S1 in columns 6 (Chalky) and 7 (Non-chalky). To be able to evaluate segmentation performance and to compare the Grad-CAM approach with Mask R-CNN, we also labeled the 660 chalky grains in terms of chalkiness area (represented as a polygon).

Similarly, out of 13,101 unpolished grains, 4085 grains were labeled as chalky and 9,016 grains were labeled as non-chalky. The exact numbers of chalky and non-chalky grains in each of the 108 high-resolution images of unpolished rice are shown in Additional file 4: Table S2 in columns 7 (Chalky) and 8 (Non-chalky). We note that many of the 36 possible genotype/tiller/condition combinations have a small number of chalky grains (or do no have any chalky grain at all). Specifically, 12 combinations corresponding to genotypes CO-39 and Kati contain 4085 chalky grains and 1299 non-chalky grains, while the remaining 24 combinations contain 151 chalky grains and 7717 non-chalky grains. Thus, we used only the 12 chalky prevalent combinations for training, tuning and evaluating the models designed in this study. Twenty chalky grain images from each of these 12 combinations (for a total of 240 images) were used as test set. To estimate the chalkiness segmentation performance on unpolished rice, the 240 test images were labelled also in terms of chalkiness area using polygons. We did not label all the chalky images in terms of chalkiness area due to the cost associated with this annotation. The number of images labeled as chalky and non-chalky and also the number of chalky images annotated in terms of chalkiness area are summarized in Table 1.

**Table 1:** Statistics on manual image annotation, specifically, the number of images labeled as chalky and non-chalky, and also the number of chalky images annotated in terms of chalky area, for polished images, and unpolished images from 12 chalky combinations, respectively

Set of seeds	Chalky	Non-chalky	Total	Chalky area
Polished	660	995	1645	660
Unpolished (12)	3934	1299	5233	240

#### Training, development and test datasets

To train, fine-tune and evaluate our models, we created training, development and test datasets for both polished and unpolished (12) grain images. In the case of polished grain images, for each grain size and chalkiness degree combination, we aimed to use one of the three replicates for training, another one for development and the last one for testing. However, to ensure a larger number of images in the training set (which is common practice in machine learning), we moved some of the instances from the development and test replicates/subsets to the training subset, so that the final distribution of the data split was approximately 2:1:1. In the case of unpolished seed images, for each genotype, tiller and condition combinations, we used a similar procedure to split the three replicates into training training, development and test subsets. The specific distribution of chalky/non-chalky grain images in the training/development/test subsets is shown in Table 2 for both polished and unpolished rice. It should be noted that our splitting process ensures that the training subset contain all types of grains considered and there is no grain that belongs to at least two subsets. We used the training subsets to train the models (both Grad-CAM networks for binary chalky/non-chalky classification and the Mask R-CNN networks for chalkiness segmentation). We used the development subsets to fine-tune hyper-parameters for the models. Finally, the performance of the models is evaluated on the test subsets. The subsets are made publicly available to ensure reproducibility and to enable further progress in this area.

**Table 2:** Distribution over Training/Development/Test subsets

Set of seeds	Training		Development		Test		Total
	Chalky	Non-chalky	Chalky	Non-chalky	Chalky	Non-chalky	
Polished	326	497	168	243	166	245	1645
Unpolished (12)	1856	830	483	229	1595	240	5233

### Experimental setup

In this subsection, we state several research questions that we aim to address and describe the experiments performed to answer these questions. We also discuss the metrics used to evaluate the models trained in our experiments and the hyper-parameters that were fine-tuned to obtain the most accurate models.

#### Research questions

We aim to answer the following research questions (RQ):

RQ1: Among different CNN networks used as the backbone in the Grad-CAM models for polished rice, what network is the best overall in terms of chalky/non-chalky classification performance versus time and memory requirements? Also, what network is the best overall in terms of chalkiness segmentation?
RQ2: How does the Grad-CAM weakly supervised approach to chalkiness segmentation compare with the Mask R-CNN segmentation approach to chalkiness detection in polished rice?
RQ3: What is the performance of the Grad-CAM models for unpolished rice? What is the performance of the polished rice models when used to make predictions on unpolished rice? Does the performance improve if we fine-tune the polished rice models with unpolished rice?

#### Experiments

To answer RQ1, we trained Grad-CAM models with several CNN networks as backbone, including variants of AlexNet, DenseNet, ResNet, SqueezeNet, VGG and EfficientNet pre-trained on ImageNet. We compared the models in terms of classification performance, memory and time requirements. We also identified the best model/network for each type of architecture. Subsequently, we study the variation of those best models with respect to the layer used to generate the heatmaps and the threshold used to binarize the heatmaps when calculating the average Intersection-over-Union (IoU). The goal is to identify the best overall layer and threshold for each type of network. The best models (with the best layer and threshold) are used to evaluate the localization accuracy, both quantitatively and qualitatively, for chalkiness detection in polished rice. To answer RQ2, we also trained Mask R-CNN models (with the default ResNet-101 as backbone) and compared them with the best weakly supervised Grad-CAM approach. Finally, to answer RQ3, we first trained and evaluated a Grad-CAM model (with ResNet-101 as backbone) on unpolished rice. We compared the performance of the resulting model with the performance of a model trained on polished rice and also with the performance of the polished rice model fine-tuned on unpolished rice.

#### Evaluation metrics

**Fig. 4:**
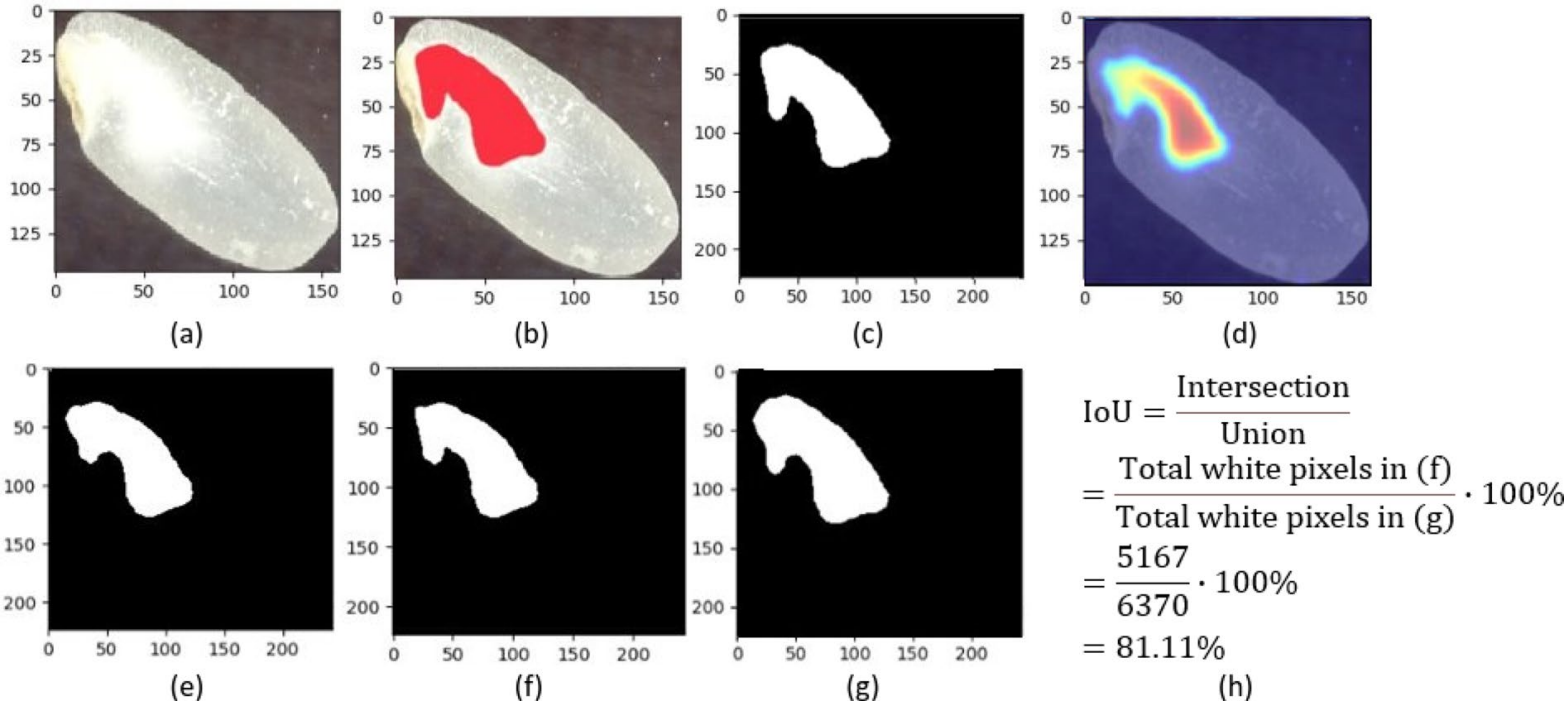
Calculating the IoU between binarized ground truth and prediction: **a** chalky seed; **b** corresponding ground truth chalkiness area; **c** binarized ground truth area; **d** predicted chalkiness area; **e** corresponding predicted binarized area; **f** intersection between the binarized ground truth (**c**) and prediction (**e**): the number of white pixels in the intersection is 5167; **g** union between the binarized ground truth (**c**) and prediction (**e**): the number of white pixels in the union is 6370; **h** Calculation of IoU

We evaluated the performance of the Grad-CAM approach along two main dimensions. First, we evaluated the ability of the approach to correctly classify seeds as chalky and non-chalky using standard classification metrics such as accuracy, precision, recall and F1 measure. Second, we evaluated the ability of the approach to perform chalkiness segmentation (i.e., the ability to identify the chalky area in the chalky seed images) using standard segmentation metrics. Specifically, we calculated average IoU [89], as well as localization accuracy and ground truth known (GT-known) localization accuracy [90] for the chalky class. Figure 4 illustrates the process of calculating IoU between the ground truth mask for the chalkiness area and the predicted chalkiness mask. As opposed to classification accuracy, which considers a prediction to be correct if it exactly matches the ground truth label, the localization accuracy considers a prediction to be correct if both the image label and the location of the detected object are correct. For the location of the object to be correct, the object mask needs to have more than 0.5 overlap with the ground truth mask. The overlap is measured as the IoU. In our case, we calculated the localization accuracy for the chalky class as the fraction of seed images for which the predicted mask for the chalky area has more than 50% IoU with the ground-truth mask. We also calculated the GT-known localization accuracy, which eliminates the influence of the classification results, as it considers a prediction to be correct when the IoU between the ground truth mask and estimated mask (in our case, for the chalky class seed images) is 0.5 or more.

#### Hyper-parameter tuning

Deep learning models, in general, and the ResNet, VGG, SqueezeNet, DenseNet EfficientNet networks, in particular, have many configurable hyper-parameters. We tuned several hyper-parameters shown to affect the performance of all models. More specifically, we tuned the batch size used in gradient descent to control the number of rice seeds processed before updating the internal model weights. Furthermore, we tuned the learning rate which controls how much we are adjusting the network weights with respect to the gradient of the loss function. The specific values that we used to tune the batch size were 16, 32 and 64. The values used to tune the learning rate were 0.1, 0.01, 0.001, 0.0001 and 0.00001. For each network, the best combination of parameters was selected based on the F1 score observed on the validation subset. Each model was run for 200 epochs and the best number of epochs for a model was also selected based on the validation subset. Overall, our hyper-parameter tuning process revealed that the performance did not change significantly with the parameters considered. All the models were trained on Amazon Web Services (AWS) p3.2xlarge instances. According to AWS^2^, the configuration of the p3.2xlarge instance is as follows: 1 GPU, 8 vCPUs, 61 GiB of memory, and up to 10 Gbps network performance.

As opposed to the models used as backbone for the Grad-CAM approach, the Mask R-CNN network with ResNet-101 as backbone could only be trained with a batch size of 8 images on AWS p3.2xlarge instances. The same learning rate values as for the CNN networks were used for tuning. However, this network was trained for a total of 600 epochs, as opposed to just 200 epochs for the other models. No other hyper-parameters specific to Mask R-CNN network were fine-tuned.

## Results and discussion

### Chalkiness classification and detection in polished rice using Grad-CAM models

#### Chalkiness classification in polished rice

Table 3 shows classification results for a variety of network architectures (and variants within one type of architecture) that were used as backbone for the Grad-CAM models. Specifically, we experimented with variants of the DenseNet, ResNet, SqueezeNet, VGG, and EfficientNet architectures. All the variants that we used have models pre-trained on ImageNet, which allowed us to perform knowledge transfer and train weakly supervised models for chalkiness detection with a relatively small number of chalky/non-chalky seed images. Only models that we could train on AWS p3.2xlarge instances were included in the table to allow for a fair comparison in terms of training time. Each model is trained and fine-tuned on the training and development subsets consisting of polished rice seed images. Performance is reported in terms of overall accuracy and also precision, recall and F1 measure for both the chalky and non-chalky classes. The best results for one type of architecture are highlighted with bold font. For each model included in Tables 3, 4 shows the training time (seconds), number of parameters, and size (MB) of the models versus the classification accuracy of the model.

**Table 3:** Classification results on polished rice with various networks as backbone in the weakly supervised Grad-CAM approach

Model	Acc.(%)	Chalky			Non-chalky		
		Pre.(%)	Rec.(%)	F1(%)	Pre.(%)	Rec.(%)	F1(%)
**DenseNet-121**	**95.61**	**94.58**	94.58	**94.58**	96.31	**96.31**	**96.31**
DenseNet-161	95.12	92.44	**95.78**	94.08	**97.06**	94.67	95.85
DenseNet-169	94.63	92.86	93.98	93.41	95.87	95.08	95.47
ResNet-18	94.63	94.44	92.17	93.29	94.76	96.31	95.53
ResNet-34	94.15	93.29	92.17	92.73	94.72	95.49	95.10
ResNet-50	94.88	**95.03**	92.17	93.58	94.78	**96.72**	95.74
**ResNet-101**	**95.12**	93.45	**94.58**	**94.01**	**96.28**	95.49	**95.88**
ResNet-152	94.88	93.94	93.37	93.66	95.51	95.90	95.71
**SqueezeNet-1.0**	**95.12**	**93.45**	94.58	**94.01**	96.28	**95.49**	**95.88**
SqueezeNet-1.1	94.39	91.33	**95.18**	93.22	**96.62**	93.85	95.22
VGG-11	94.88	**93.94**	93.37	93.66	95.51	**95.90**	95.71
VGG-13	94.39	92.31	93.98	93.13	95.85	94.67	95.26
**VGG-16**	**95.12**	92.94	95.18	**94.05**	96.67	95.08	**95.87**
VGG-19	94.15	90.34	**95.78**	92.98	**97.01**	93.03	94.98
EfficientNetB0	95.13	93.98	93.98	93.98	95.92	95.92	95.92
EfficientNetB1	95.13	94.51	93.37	93.94	95.55	96.33	95.93
EfficientNetB2	93.67	90.23	**94.58**	92.35	**96.20**	93.06	94.61
EfficientNetB3	95.13	95.06	92.77	93.90	95.18	96.73	95.95
**EfficientNetB4**	**95.38**	**96.82**	91.57	**94.12**	94.49	**97.96**	**96.19**
EfficientNetB5	93.67	91.67	92.77	92.22	95.06	94.29	94.67
EfficientNetB6	94.16	92.77	92.77	92.77	95.10	95.10	95.10

The number following a network’s name denotes the number of layers in the network (as in DenseNet-121 or ResNet-101) or the version of the network (as in SqueezeNet-1.0 or EfficientNetB0). Performance is reported in terms of Accuracy (Acc.), Precision (Pre.), Recall (Rec.) and F1 measure (F1). Precision, Recall and F1 measure values are reported separately for the Chalky and Non-Chalky classes. All models are trained/tuned/evaluated on the same training/development/test splits. The results reported are obtained on the test set. The best performance for each type of model for each metric is highlighted using bold font

**Table 4:** Classification networks: training time and model size

Model	Training time (s)	Number of parameters	Size (MB)	Acc. (%)
**DenseNet-121**	1522.88	6955906	28.4	**95.61**
DenseNet-161	2157.04	26,476,418	107.1	95.12
DenseNet-169	1306.20	12,487,810	50.9	94.63
ResNet-18	546.77	11,177,536	44.8	94.63
ResNet-34	719.41	21,285,696	85.3	94.15
ResNet-50	1011.85	23,512,128	94.4	94.88
**ResNet-101**	1668.41	42,504,256	170.6	**95.12**
ResNet-152	2172.97	58,147,904	233.4	94.88
**SqueezeNet-1.0**	533.15	736,450	3.0	**95.12**
SqueezeNet-1.1	481.53	723,522	2.9	94.39
VGG-11	2382.44	128,774,530	515.1	94.88
VGG-13	2641.00	128,959,042	515.9	94.39
**VGG-16**	2745.00	134,268,738	537.1	**95.12**
VGG-19	3079.89	139,578,434	558.4	94.15
EfficientNetB0	1198.53	4,052,126	33.0	95.13
EfficientNetB1	2243.48	6,577,794	53.4	95.13
EfficientNetB2	1882.26	7,771,380	62.9	93.67
EfficientNetB3	2696.21	10,786,602	87.1	95.13
**EfficientNetB4**	3476.74	17,677,402	142.3	**95.38**
EfficientNetB5	3584.68	28,517,618	229.1	93.67
EfficientNetB6	4946.95	40,964,746	328.3	94.16
Mask R-CNN	14863.00	42,504,256	255.9	N/A

The number following a network’s name denotes the number of layers in the network (as in DenseNet-121 or ResNet-101) or the version of the network (as in SqueezeNet-1.0 or EfficientNetB0). All models are trained on AWS p3.2xlarge instances. The training time it took to train each model for 200 epochs is reported in seconds (s). Model complexity is reported as the number of trainable parameters of the model, as well as the size of the model in MB. The accuracy of each model is also shown, and the best accuracy (Acc.) obtained for each type of model is highlighted in bold font

As can be seen from Table 3, the overall classification accuracy varies from 93.67% (for EfficientNetB2 and EfficientNetB5) to 95.61% (for DenseNet-121). The DenseNet-121 model, which has the highest classification accuracy, also has the highest F1 measure for both chalky and non-chalky classes, although there is at least one competitive variant for each architecture type, e.g., ResNet-101 for ResNet, SqueezeNet-1.0 for SqeezeNet, VGG-16 for VGG, and EfficientNetB4 for EfficientNet. Furthermore, the DenseNet-121 model has a relatively small size (28 MB) and average training time (approximately 1500 s). Surprisingly, the SqueezeNet architecture, which is highly competitive in terms of performance, has the smallest size (3.0/2.9 MB for SqueezeNet-1.0/SqueezeNet-1.1, respectively) and smallest training time (approximately 500 s). The VGG models have the largest size (more than 500 MB) and relatively large training time (in the range of 2400 to 3000 s), and the best EfficientNet variant (EfficientNetB4) has moderate size (approximately 140 MB) but relatively large training time (approximately 3500 s). Finally, the ResNet-101 variant, which is the best in the ResNet group, has moderate size (170 MB) and training time (close to 1700 s). Based on these results, we selected one model for each type of architecture and used those selected models for further analysis.

#### Chalkiness detection in polished rice

To produce accurate detection of chalkiness area, we first studied the variation of the average IoU with respect to the layer used to generate the heatmaps and the threshold, *T*, used to binarize the heatmaps when calculating the IoU. The best layer/threshold combination was selected independently for each type of network using both qualitative and quantitative evaluations. Based on preliminary visual inspection of the heatmaps, we observed that heatmaps corresponding to lower level layers in a network result in better approximations of the chalkiness area, possibly because the progressive down-sampling along the convolutional layers of the backbone CNN makes it hard to precisely recover the chalkiness information from the higher level feature maps [91]. Therefore, for each type of network, we evaluated a lower-level layer (e.g., layer1_2_conv2 for ResNet-101), two intermediate layers (e.g., layer2_0_conv2 and layer3_1_conv2 for ResNet-101), and one high-level layer (e.g., layer4_1_conv3 for ResNet-101). The threshold, *T*, varied from $10\%$ to $80\%$ in increments of 10. We focused our analysis on ResNet-101 moving forward as this network produced the best segmentation results overall. Table 5 shows the variation of performance (i.e., average IoU over the set of chalky seed images) with the layer and the threshold for ResNet-101.

**Table 5:** Variation of the Average IoU (%) with the layer and threshold used for ResNet-101

Layer	T = 20%	T = 30%	T = 40%	T = 50%	T = 60%	T = 70%	T = 80%
layer1_2_conv2	0.20	9.90	18.41	26.08	37.53	18.55	18.55
**layer2_0_conv2**	3.81	19.86	31.53	44.90	**68.11**	18.55	18.55
layer3_1_conv2	1.77	9.59	18.92	28.22	41.59	18.55	18.55
layer4_1_conv3	0.15	10.26	15.43	21.10	29.68	18.55	18.55

The layer is used to generate the heatmaps and the threshold *T* is used to binarize the heatmaps (e.g., $T=20\%$ means that only pixels with values at least $20\%$ of the max pixel value in the image are included in the binary mask). The layers were sampled to include a low-level layer (layer1_2_conv2), a high-level layer (layer4_1_conv3) and two intermediate layers (layer2_0_conv2 and layer3_1_conv2) that showed good results based on a qualitative inspection of the maps. The threshold *T* is varied from $20\%$ to $80\%$ in increments of 10. The best result and the corresponding layer and threshold are highlighted in bold font

As shown in Table 5, for ResNet-101 we obtained better performance with a lower-intermediate layer (layer2_0_conv2) as opposed to a higher layer as reported in other studies [66, 68], and a threshold of $T=60\%$ of the highest pixel value, which is larger than the standard $T=15\%$ [66] or $T=20\%$ [68] thresholds frequently used in prior studies. A similar pattern is observed in terms of threshold with the other four networks. As for the layer, the DenseNet-121 and VGG-16 give the best results using a lower layer, while SqueezeNet-1.0 and EfficientNetB4 networks give best results with a higher layer. More details can be seen in Additional file 5: Table S3.

**Fig. 5:**
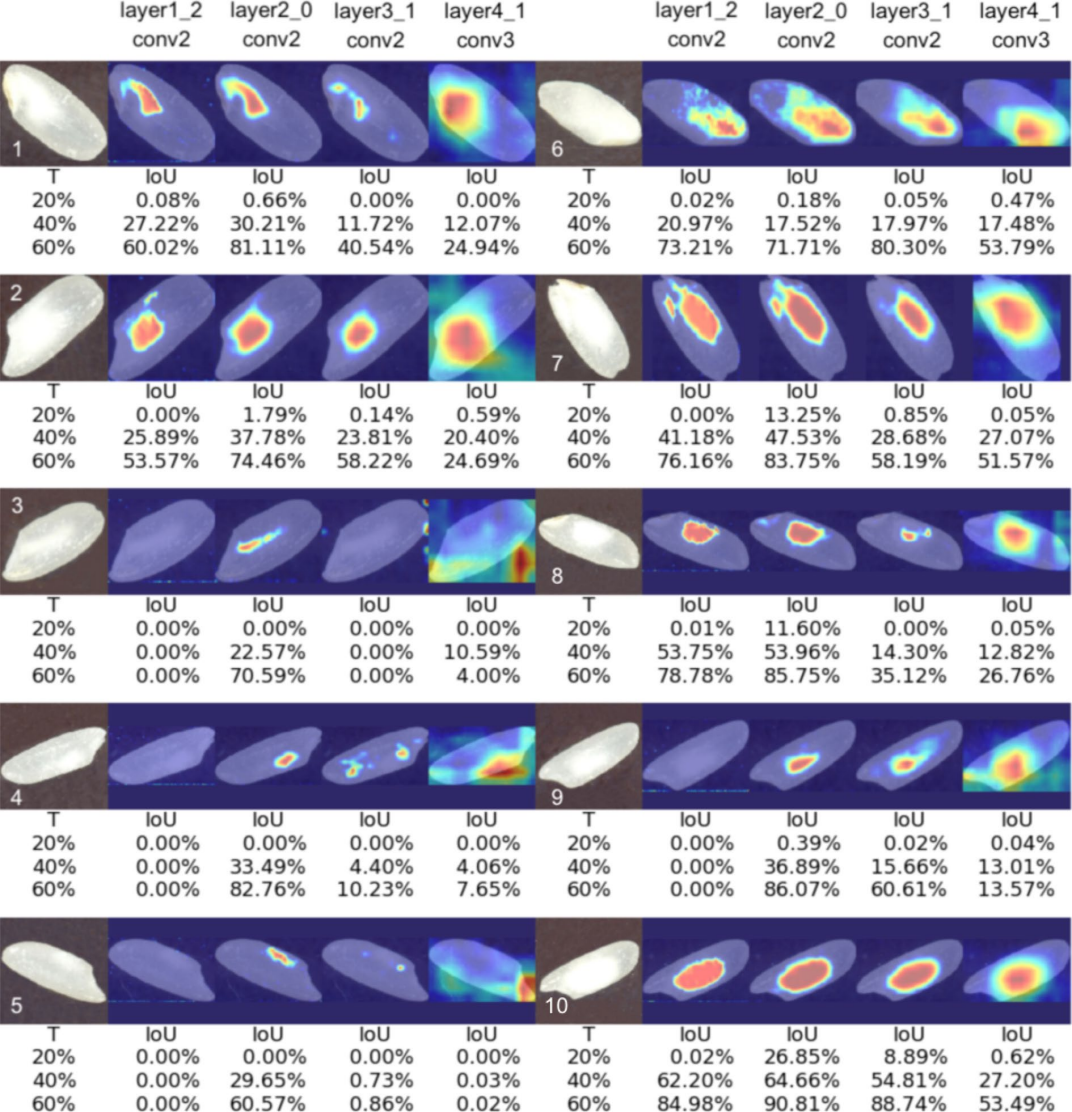
Examples of Grad-CAM (ResNet-101) heatmaps for 10 sample chalky seed images (5 on the left side and 5 on the right side). For each seed, heatmaps corresponding to the following four layers are shown: (1) ResNet101 layer1_2_conv2; (2) ResNet101 layer2_0_conv2; (3) ResNet101 layer3_1_conv2; (4) ResNet101 layer4_1_conv3. The IoU values obtained for three thresholds T (20%, 40% and 60%, respectively) are shown under each heatmap

**Fig. 6:**
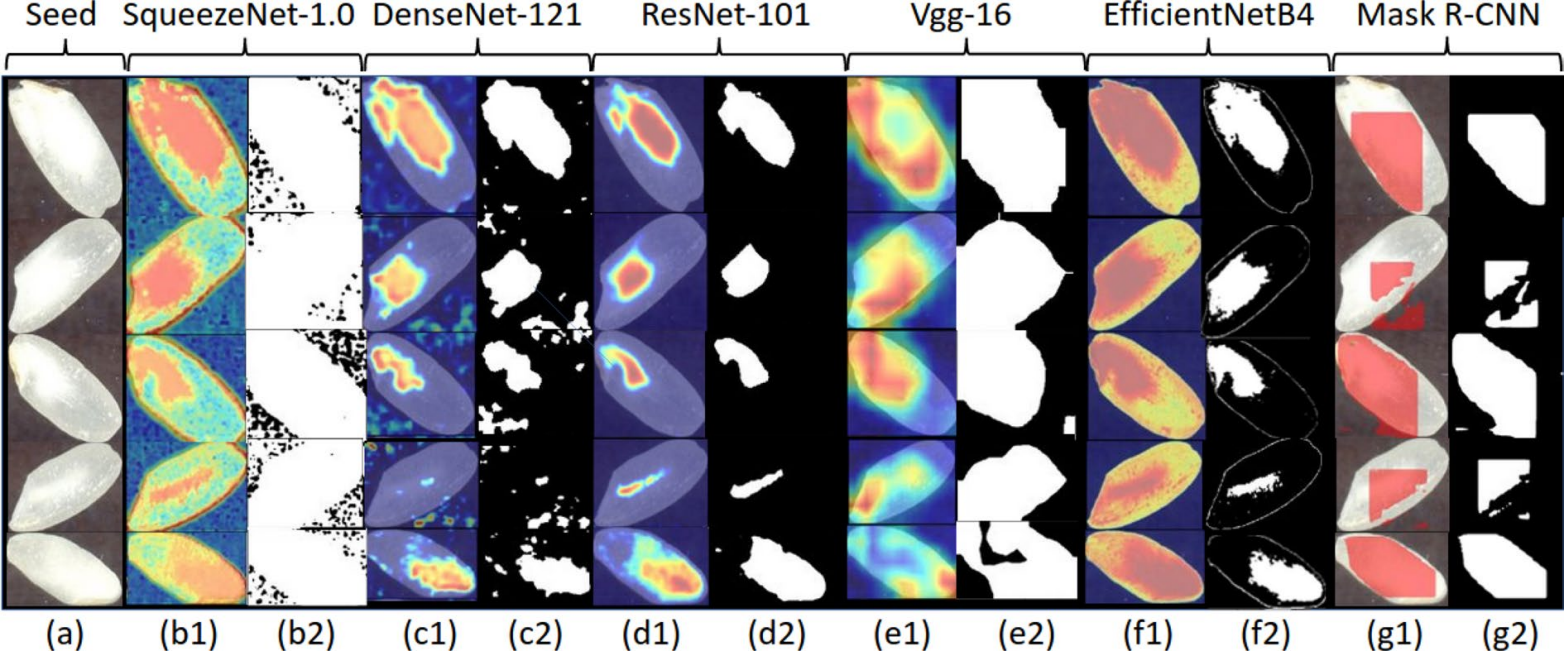
Examples of Grad-CAM heatmaps and corresponding binarized chalkiness masks. (a) Five sample chalky seed images; (b1) SqueezeNet-1.0 Heatmaps; (b2) SqueezeNet-1.0 Masks; (c1) DenseNet-121 Heatmaps; (c2) DenseNet-121 Masks; (d1) ResNet-101 Heatmaps; (d2) ResNet-101 Masks; (e1) VGG-19 Heatmaps; (e2) VGG-19 Masks; (f1) EfficientNetB4 Heatmaps; (f2) EfficientNetB4 Masks; (g1) Mask R-CNN Original Masks ; (g2) Mask R-CNN Binary Masks

To gain more insights into the heatmap layer and threshold, Fig. 5 shows qualitative and quantitative results obtained with Grad-CAM using ResNet-101 as backbone for 10 sample seed images in the test dataset when considering three thresholds ($20\%$, $40\%$, $60\%$) and four convolution layers. As can be seen in the figure, seeds with a larger chalky area (e.g., seeds 6 and 10) are less sensitive to the layer chosen, i.e., several layers produce heatmaps with high IoU scores. However, for seeds with a smaller or narrow chalky area, the results are more sensitive to the layer selected and the best results are obtained with the intermediate layer, layer2_0_conv2. Another observation that can be made from Fig. 5 is that, overall, the lower layers tend to have sharper boundaries as opposed to the higher levels that have softer boundaries, making it harder to find a good threshold. This may be due to the fact that higher levels in the network correspond to lower dimensional feature maps, which no longer preserve boundary details when interpolated back to higher dimensions. Additional files 6, 7, 8: Figs. S3–S5 show similar quantitative and qualitative results produced by SqueezeNet-1.0, DenseNet-121 and VGG-16 networks, respectively, on the same 10 seeds shown in Fig. 5. Despite the good classification results obtained with these networks, the heatmaps show lighter colors and softer boundaries for the chalkiness area and overall poor chalkiness detection results as compared with the results of ResNet-101. A better understanding regarding this can be gained from Fig. 6 which shows a side-by-side comparison of the heatmaps produced by different networks and the corresponding binarized chalkiness masks. The masks obtained with Mask R-CNN are also shown.

The same conclusions regarding the superiority of ResNet-101 for chalkiness segmentation are supported by a quantitative evaluation of the networks in terms of localization metrics computed over the whole test set. The results of this evaluation are shown in Table 6 for the best performing models for each type of architecture considered as backbone (DenseNet-121, ResNet-101, SqueezeNet-1.0, VGG-19, and EfficientNetB4). For each network, the specific convolution layer and the threshold used to produce the results are shown in the last two columns of the table, respectively. The results obtained with the Mask R-CNN network, which has ResNet101 as its backbone, are also shown in Table 6. As can be seen, the best results are obtained using the ResNet-101 network (for all metrics considered), while the next best results are obtained with DenseNet-121. Among the weakly supervised Grad-CAM networks, the ones that have SqueezeNet-1.0 and VGG-16 as backbones, produce the worst results. The results of the Mask R-CNN network are extremely poor when compared with the results of the Grad-CAM with ResNet-101, DenseNet-121 and EfficientNetB4 backbones but they are better than those of the Grad-CAM with SqueezeNet-1.0 and VGG-16 as backbones. This shows that the weakly supervised approach is more effective for the chalkiness detection/segmentation problem in addition to being less laborious in terms of data labeling, as compared to the Mask R-CNN segmentation approach.

**Table 6:** Chalkiness Segmentation: results of the weakly supervised Grad-CAM approach with the best performing classification models as backbone

Model	GT-known Loc. Acc. (%)	Loc. Acc. (%)	Avg. IoU (%)	Layer	T (%)
Grad-CAM (DenseNet-121)	51.20 = 085/166	51.20 = 085/166	47.44	Features_ denseblock2_ denselayer7_ conv2	60
Grad-CAM (ResNet-101)	84.34 = 140/166	83.13 = 138/166	68.11	Layer2_0_ conv2	60
Grad-CAM (SqueezeNet-1.0)	15.06 = 025/166	0 = 00/166	31.01	Features_12 _expand1x1	60
Grad-CAM (VGG-16)	7.23 = 012/166	7.23 = 012/166	24.92	Features_ module_5	60
Grad-CAM (EfficientNetB4)	28.92 = 048/166	28.92 = 048/166	35.40	Stem _conv	50
Mask R-CNN (ResNet-101)	18.67 = 031/166	N/A	29.63	N/A	N/A

The results of Mask R-CNN with ResNet-101 as backbone are also shown. Only the 166 chalky seed images in the test set were used for chalkiness segmentation evaluation. Performance is reported using the following metrics (as applicable): Ground-Truth Localization Accuracy (GT-known Loc. Acc.), which represents the fraction of ground-truth chalky seed images with $\text{ IoU } \ge 0.5$; Localization Accuracy (Loc. Acc.), which represents the fraction of ground-truth chalky images, with $\text{ IoU } \ge 0.5$, correctly predicted by the model; Average IoU (Avg. IoU), which represents the average IoU for the set of chalky seed images. To calculate the IoU, the mask of the predicted chalkiness is obtained using a threshold $T=60\%$ of the maximum pixel intensity. The last two columns show the layer that was used for generating the heatmap and the threshold used to binarize the heatmap when calculating IoU, respectively

To understand if the results obtained with Grad-CAM and ResNet-101 can be further improved with an alternative localization approach, we compared Grad-CAM with Grad-CAM++ and Score-CAM (using ResNet-101 as the backbone CNN). The results of the comparison are shown in Table 7 and show that Grad-CAM consistently outperforms its Grad-CAM++ variant and the Score-CAM approach. While this may seem surprising at first, we note that Grad-CAM++ has been designed to handle multiple occurrences of an object in an image. However, in our task, chalkiness is a concept with soft boundaries and doesn’t present multiple occurrences. Thus, Grad-CAM++ may highlight additional regions that are not representative of chalkiness, as marked by human annotators. As for Score-CAM, this approach has been shown to find firmer, less fuzzy localizations of the objects of interests. However, as chalkiness inherently has relatively fuzzy, soft boundaries, Score-CAM usually highlights a smaller area as compared with the manual annotations, resulting in worse overall results compared with Grad-CAM, although better than Grad-CAM++. Additional file 9: Fig. S6 shows the heatmaps found by the three approaches (Grad-CAM, Grad-CAM++ and Score-CAM) by comparison with the manually annotated chalkiness area for four sample seeds. The heatmaps support our conclusions regarding Grad-CAM++ and Score-CAM results (shown in Table 7).

**Table 7:** Comparison between the chalkiness segmentation results of the weakly supervised approaches Grad-CAM, Grad-CAM++ and Score CAM with ResNet-101 as backbone on polished rice

Approach	GT-known Loc. Acc. (%)	Loc. Acc. (%)	Avg. IoU (%)	Layer	T (%)
Grad-CAM	**84.43**	**84.13**	**68.11**	layer2_0_conv2	60
Grad-CAM++	48.19	48.19	43.98	layer2_0_conv2	60
Score-CAM	68.07	66.87	55.02	layer2_2_conv3	60

Only 166 chalky seed images in the polished test set were used for chalkiness segmentation evaluation. Performance is reported using the following metrics: Ground-Truth Localization Accuracy (GT-known Loc. Acc.), which represents the fraction of ground-truth chalky seed images with $\text{ IoU } \ge 0.5$; Localization Accuracy (Loc. Acc.), which represents the fraction of ground-truth chalky images, with $\text{ IoU } \ge 0.5$, correctly predicted by the model; Average IoU (Avg. IoU), which represents the average IoU for the set of chalky seed images. To calculate the IoU, the mask of the predicted chalkiness is obtained using a threshold $T=60\%$ of the maximum pixel intensity. The last two columns show the layer that was used for generating the heatmap and the threshold used to binarize the heatmap when calculating IoU, respectively

### Chalkiness classification and detection in unpolished rice

Another objective of this study is to explore the applicability of the Grad-CAM approach to unpolished rice seeds and to study the transferability of the models trained on polished rice to unpolished rice (as unpolished rice seeds can be harder to annotate manually). This is important as researchers working on large breeding populations involving hundreds of lines do not obtain large sample sizes and would not have access to polish a small amount of seeds, which requires models that can effectively operate on unpolished seeds. To address this objective, we performed experiments with three models that use ResNet-101 as their backbone: (1) a model trained on polished seed images, called *polished model*; (2) a model trained on unpolished seed images, called *unpolished model*; and (3) a model originally trained on polished seed images and subsequently fine-tuned on unpolished seed images, called *mixed model*. All models were evaluated on the 240 seed images in the unpolished test set, which were manually annotated in terms of chalkiness area. These images belong to one of the 12 combinations corresponding to the Kati and CO-39 genotypes, i.e., unpolished (12) set. The training and developments sets used to train the unpolished and mixed models belong to the unpolished(12) set as well (see Table 2). Classification results for the three models are shown in Table 8, while segmentation results are shown in Table 9. As can be seen in Table 8, the mixed model performs the best overall in terms of classification metrics, although the unpolished model has similar performance for both chalky and non-chalky classes. However, as Table 9 shows, the unpolished model is by far the most accurate in terms of segmentation metrics, while the polished model is the worst.

**Table 8:** Classification results on unpolished rice when ResNet-101 is used as backbone in the weakly supervised Grad-CAM approach

ResNet-101	Acc.(%)	Chalky			Non-chalky		
		Pre.(%)	Rec.(%)	F1(%)	Pre.(%)	Rec.(%)	F1(%)
Polished	63.01	0.00	0.00	0.00	**63.01**	**100.00**	**77.31**
Unpolished	83.43	**98.50**	82.19	89.61	43.65	91.67	59.14
Mixed	**84.20**	98.08	**83.45**	**90.18**	44.77	89.17	59.61

Three models are evaluated: 1) *polished model* trained on polished rice images; 2) *unpolished model* trained on Unpolished (12); 3) *mixed model*, obtained by further training the polished model using the Unpolished (12) images. Performance is reported in terms of Accuracy (Acc.), Precision (Pre.), Recall (Rec.) and F1 measure (F1). Precision, Recall and F1 measure values are reported separately for the Chalky and Non-Chalky classes. All three models are evaluated on the test subset corresponding to the Unpolished (12) rice images. The best performance for each type of model for each metric is highlighted using bold font

**Table 9:** Chalkiness segmentation results of the weakly supervised Grad-CAM approach with ResNet-101 as backbone on unpolished rice

Grad-CAM (ResNet-101)	GT-known Loc. Acc. (%)	Loc. Acc. (%)	Avg. IoU (%)	Layer	T (%)
polished model	7.92 = 019/240	7.92 = 19/240	26.79	layer2_0_ conv2	60
unpolished model	63.75 = 153/240	63.75 = 153/240	51.76	layer2_0_ conv2	60
mixed model	20.42 = 049/240	20.42 = 049/240	29.91	layer2_3_ conv2	60

Only 240 chalky seed images in the Unpolished (12) test set were used for chalkiness segmentation evaluation. Performance is reported using the following metrics: Ground-Truth Localization Accuracy (GT-known Loc. Acc.), which represents the fraction of ground-truth chalky seed images with $\text{ IoU } \ge 0.5$; Localization Accuracy (Loc. Acc.), which represents the fraction of ground-truth chalky images, with $\text{ IoU } \ge 0.5$, correctly predicted by the model; Average IoU (Avg. IoU), which represents the average IoU for the set of chalky seed images. To calculate the IoU, the mask of the predicted chalkiness is obtained using a threshold $T=60\%$ of the maximum pixel intensity. The last two columns show the layer that was used for generating the heatmap and the threshold used to binarize the heatmap when calculating IoU, respectively

**Fig. 7:**
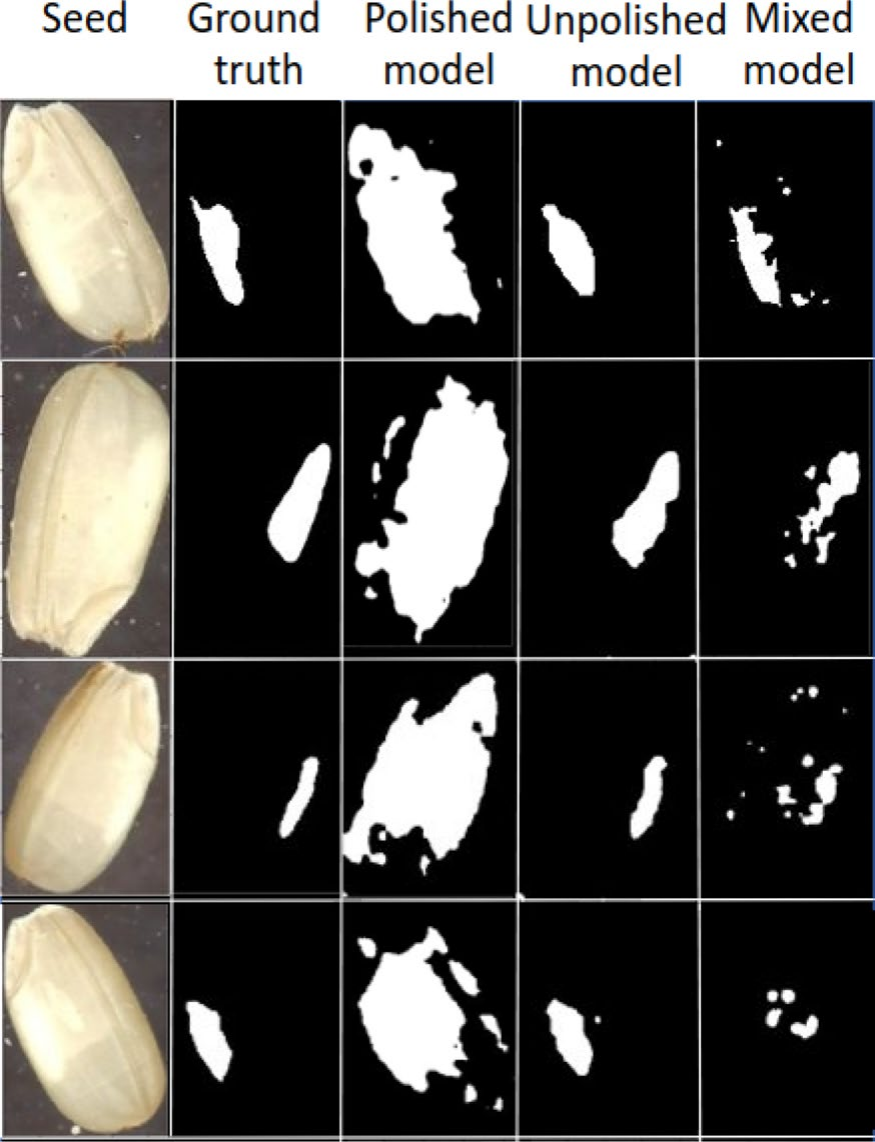
Examples of chalkiness binary masks for four unpolished rice grains. The binary masks obtained from the Grad-CAM heatmaps (with ResNet-101 as backbone) using a threshold $T=60\%$ are shown form the polished, unpolished and mixed models, respectively, by comparison with the ground truth binary mask

To visually illustrate the output of each model, Fig. 7 shows the chalkiness prediction masks of the polished, unpolished and mixed models for four unpolished seeds. The polished model largely over-estimates the chalkiness area given the opaque nature of the unpolished seeds, as opposed to the translucent appearance of the polished seeds. The mixed model improves the masks but not as much as the unpolished model that is trained specifically on unpolished rice seeds. Together, these results suggest that not much knowledge can be transferred directly from the polished images to unpolished images, as the appearance of the chalkiness is different between polished and unpolished seeds. The results can be improved with the mixed model which fine-tunes the polished models on unpolished rice, although the fine-tuned models still lag behind the models trained directly on unpolished rice. Hence, models developed using polished or unpolished grains needs to be used based on the objective with poor transferability across these two categories.

### Answers to the Research Questions and Error Analysis

As mentioned in Section "Experimental setup", we set to answer three main research questions: [RQ1] aims to identify the best Grad-CAM models for polished rice, in terms of classification and segmentation performance; [RQ2] is focused on the segmentation performance of the weakly supervised Grad-CAM approach by comparison with Mask R-CNN; and [RQ3] is focused on the performance of models for classifying unpolished rice and transferability of information from polished to unpolished rice.

To answer RQ1, we evaluated several CNN architectures in terms of classification accuracy, memory and time requirements, and also chalkiness detection performance in polished rice. While the architectures studied have comparable classification performance, the ResNet-101 network was found to be superior with respect to chalkiness detection in polished rice and has relatively small memory and time requirements. Furthermore, we compared the best weakly supervised Grad-CAM models with the Mask R-CNN segmentation model to answer RQ2 and found that the Grad-CAM models performed better than Mask R-CNN, which needs more expensive pixel level annotation. Overall, the chalkiness detection results obtained for polished rice are remarkably good, with an average IoU of 68.11%, GT-known accuracy of 83.34% and localization accuracy of 83.13%. Finally, to answer RQ3, we used Grad-CAM models trained on polished rice, unpolished rice, and a mix of polished and unpolished rice and evaluated them on unpolished rice. When studying the transferability of the models trained on polished rice to unpolished rice, we found that fine-tuning on unpolished rice is necessary. In fact, models trained directly on the unpolished rice performed the best in our study. More specifically, our evaluation on unpolished rice grain images showed that the best model trained directly with unpolished rice had an average IoU of 51.76%, while both the GT-known accuracy and the localization accuracy were 63.75%. It is not surprising that the models perform better on polished rice as chalkiness is easier to detect after the interfering aluerone layer is removed through milling.

While the use of the Grad-CAM approach for rice chalkiness segmentation was extremely successful, one challenge that we encountered was the tuning of the layer to be used for generating the heatmaps as well as the threshold for producing the binary masks for chalkiness area. Our goal was to find a good overall layer and threshold for a model to avoid the pitfall of tuning the threshold for each type of rice seed. Our analysis showed that a lower layer generally results in better chalkiness detection. One explanation for this is that higher levels undergo more extensive down-sampling (through successive applications of pooling layers) and this causes loss of information that cannot be recovered in the chalkiness heatmaps. Regarding the threshold for binarization, our results showed that a higher threshold (e.g., $T=60\%$) produces better overall results. One possible reason for the higher threshold may be given by the fact that our images have relatively low contrast between the chalky area and its neighboring area, as compared to other segmentation tasks for which weakly supervised approaches have been used. However, a threshold of 60% has also been used for binarizing gray images, e.g. fingerprint images [92] or textile pilling images [93], which are similar in nature to our chalkiness images.

**Fig. 8:**
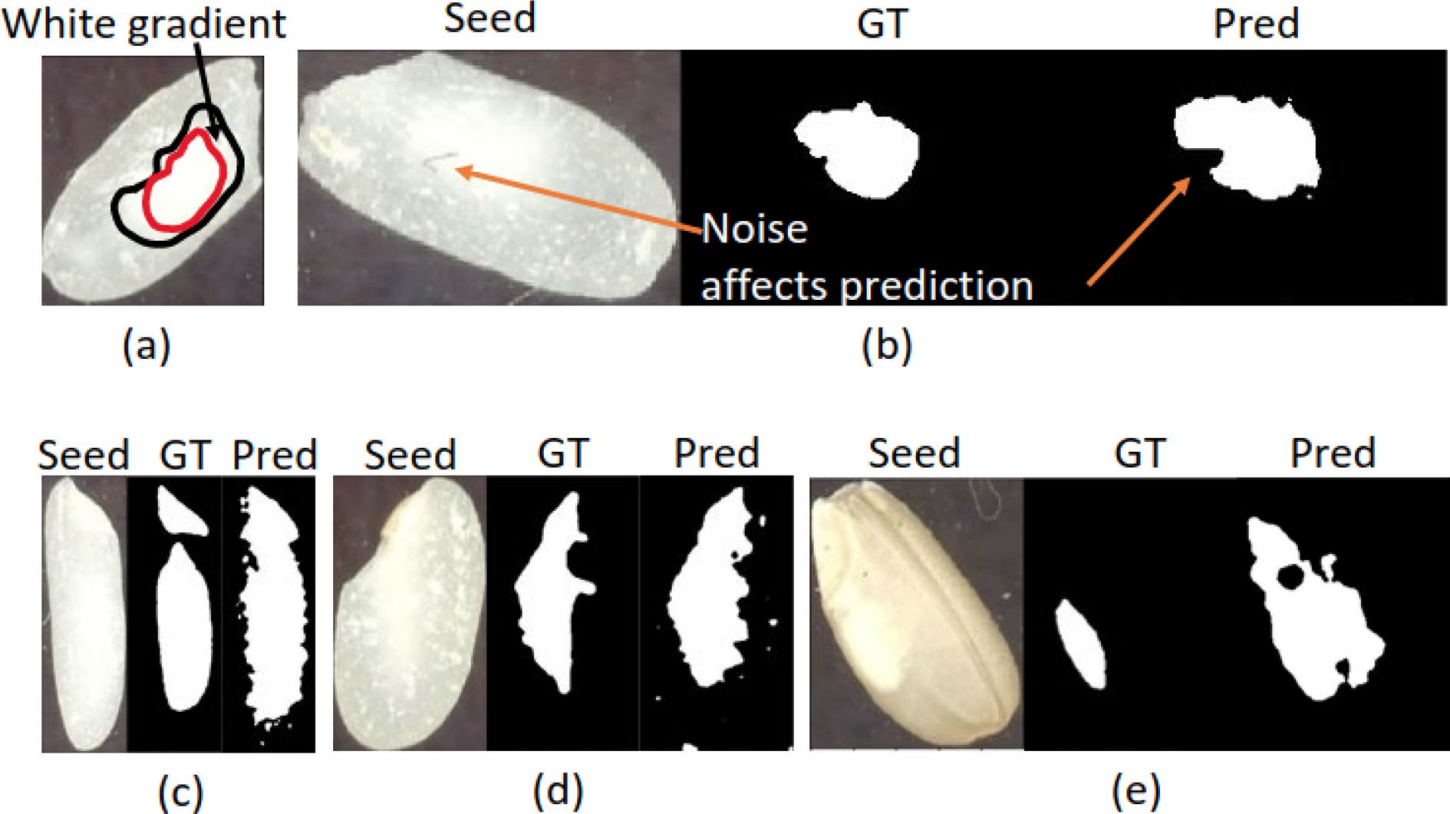
Sources of errors for the Grad-CAM models. Images (**a**–**d**) correspond to polished rice, while image (**e**) corresponds to unpolished rice. The sources of error can be summarized as: **a** Inconsistencies in the way chalkiness is manually annotated, due to the white gradient nature of chalkiness; **b** Scratches or marks (referred as noise) on the chalkiness area can be interpreted as non-chalkiness; **c** Irregular chalkiness shape makes it hard to annotate chalkiness very precisely; **d** Abrasion stains can be recognized as chalkiness (white dots on the right in the figure); **e** Irregular shape and fuzzy boundaries affect the ground truth annotations and the predictions in unpolished rice as well

Error analysis of the polished models revealed several sources of errors that lead to disagreement between model predictions and ground truth annotations. Such sources are illustrated in Fig. 8 and include: (a) inconsistencies in the way chalkiness is manually annotated due to the soft/fuzzy boundaries of chalkiness (as opposed to binary chalky versus non-chalky boundaries); (b) scratches or marks (referred to as noise) on the chalkiness area are interpreted as non-chalkiness and lead to mismatches with the ground truth annotations in terms of IoU metric; (c) irregular chalkiness shapes also make it hard to annotate chalkiness very precisely; (d) abrasion stains that are recognized as chalkiness (white dots on the right in the figure) despite the fact that the Grad-CAM model uses deeper feature maps that presumably miss some “details”; (e) irregular shape and fuzzy boundaries affect the ground truth annotations and consequently the predictions in unpolished rice as well. Despite such errors, we found that the best Grad-CAM model for unpolished rice, trained on the Kati and CO-39 genotypes, can generalize well to unpolished rice grains from the other genotypes included in the biological experiment. Additional file 10: Fig. S7 shows the prediction results of the unpolished model on 12 rice grains randomly selected from the genotypes not used in the training, together with their manual annotations. When analyzing images predicted as false positives by the model with ResNet-101 as backbone, we observed that the main reason for the model to predict non-chalky images as chalky is the presence of larger abrasion stains or damaged seed ends that are recognized as chalkiness, although not considered to be chalkiness by manual annotators. Some examples of false positive seed images, together with their corresponding chalkiness heatmaps produced by Grad-CAM are shown in Additional file 11: Fig. S8.

### Tool availability and time requirements

In terms of time requirements, our experiments showed the average time for training a ResNet-101 model on an EC2 p3-2xlarge instance available from AWS is 1668.41 s, as shown in Table 4, and no human intervention is required during that time. Once the model is trained, the average time to predict the label of a new image and create a chalkiness heatmap is less than 1 s using an EC2 p2-xlarge instance. Given these time requirements and assuming that thousands of images need to be annotated for genetic mapping studies, our models could be extremely cost-effective and help save significant human efforts and time that would otherwise be invested in the manual annotation.

### Development of rice with less chalk under future hotter climate

Quantifying rice chalkiness rapidly and accurately continues to be a limitation for capturing the degree of chalkiness across a wide range of genetic backgrounds due to the lack of a high throughput phenotyping tool. Developing such a tool is important and timely as the proportion of chalky grains are bound to increase under warming scenarios, particularly with increasing night temperatures [19, 94]. We used the tool developed based on Grad-CAM to determine the percent chalkiness area and the chalkiness score for each of the 13,101 unpolished rice grains extracted from the original scanned images. As opposed to the chalkiness area, which is obtained based on a binary map, the chalkiness score considers the intensity of chalkiness for each pixel, with red indicating greater proportion of chalk per pixel and blue indicating the least proportion of chalk per pixel (Figs. 5 and 6). Subsequently, we aggregated the percent chalkiness and the chalkiness score per sample (i.e., for each combination genotype/tiller/treatment). Using the aggregates, we analyzed differences between genotypes, tiller and treatment in terms of chalkiness in three scenarios. In scenario 1, where the chalkiness was determined using the coarse chalky versus non-chalky classification of the grains, analysis based on the number of grains with and without chalk resulted in a poor analytical resolution and failed to detect any differences or significant interaction effects (Additional file 12: Table T4). In scenario 2, analysis based on the proportion of area of chalkiness determined from the Grad-CAM binarized heatmaps improved the prediction power where apart from genotype (G) main effect, the interaction effects of HNT treatment (T) *G, G* panicle type (P), and T*G*P interaction effects were significant (Table 10). This finding indicated that the approach was able to detect the differential proportion of chalkiness in different tillers across genotypes under HNT exposure during grain-filling. Using this approach, genotypic differences in the proportion of accumulation of chalkiness were observed with IR1561 and WAS-174 which recorded an increase of chalkiness in grains in primary and other panicles as compared to main tiller under HNT, while the same was reduced in IR-22 and Kati and was not affected in CO-39 and Oryzica (Table 10). Percent change in proportion of chalkiness under HNT in primary and other panicles compared to main panicle ranged from −0.89% in IR1561 to 122% in WAS-174. Grains from both primary and other panicles recorded an increase in proportion of chalkiness by 63 and 122%, respectively, compared to main panicle under HNT in WAS-174 (Table 10). In scenario 3, the chalkiness score was calculated using the pixel intensity in the chalkiness heatmaps produced by Grad-CAM and analysis of variance for chalkiness score revealed a significant effect of G, T*G, G*P and T*G*P further indicating an improvement in prediction potential for chalkiness among genotypes, treatments and tiller types (Table 10).

**Table 10:** Percentage chalkiness area and chalkiness score were obtained for individual seeds randomly selected across treatments and genotypes

Chalkiness (% area)	Main panicle		Primary panicle		Other panicle	
	CNT	HNT	CNT	HNT	CNT	HNT
CO-39	7.54 $\pm 0.8$	8.17 $\pm 1.0$	6.95 $\pm 0.6$	7.73 $\pm 0.3$	8.00 $\pm 1.8$	7.19 $\pm 1.1$
IR1561	13.35 $\pm 2.5$	16.22 $\pm 3.0$	8.21 $\pm 0.2$	16.37 $\pm 2.4$	8.52 $\pm 1.1$	12.35 $\pm 1.2$
IR-22	8.02 $\pm 0.8$	6.33 $\pm 0.5$	9.36 $\pm 2.2$	5.27 $\pm 0.5$	5.89 $\pm 0.9$	5.30 $\pm 0.6$
Kati	7.39 $\pm 0.7$	7.44 $\pm 0.4$	13.56 $\pm 1.2$	10.34 $\pm 2.5$	14.32 $\pm 1.9$	10.70 $\pm 2.5$
Oryzica	10.61 $\pm 1.4$	10.75 $\pm 1.8$	5.32 $\pm 0.5$	5.64 $\pm 0.6$	5.05 $\pm 0.6$	4.83 $\pm 1.4$
WAS-174	7.25 $\pm 2.0$	5.76 $\pm 1.7$	5.91 $\pm 0.4$	9.39 $\pm 2.6$	4.44 $\pm 0.8$	12.81 $\pm 1.4$

Chalkiness score	CNT	HNT	CTN	HNT	CTN	HNT
CO-39	0.07518 $\pm 0.008$	0.06827 $\pm 0.009$	0.07157 $\pm 0.004$	0.07780 $\pm 0.003$	0.07449 $\pm 0.016$	0.07119 $\pm 0.010$
IR1561	0.10415 $\pm 0.015$	0.12933 $\pm 0.026$	0.07026 $\pm 0.002$	0.14653 $\pm 0.032$	0.07006 $\pm 0.006$	0.11615 $\pm 0.012$
IR-22	0.07276 $\pm 0.003$	0.06294 $\pm 0.006$	0.09692 $\pm 0.017$	0.05694 $\pm 0.006$	0.05916 $\pm 0.012$	0.05686 $\pm 0.007$
Kati	0.09238 $\pm 0.009$	0.09252 $\pm 0.002$	0.17087 $\pm 0.017$	0.13309 $\pm 0.032$	0.16940 $\pm 0.018$	0.13586 $\pm 0.029$
Oryzica	0.14890 $\pm 0.024$	0.16370 $\pm 0.029$	0.08302 $\pm 0.011$	0.08862 $\pm 0.006$	0.07928 $\pm 0.014$	0.08246 $\pm 0.026$
WAS-174	0.09386 $\pm 0.024$	0.07700 $\pm 0.023$	0.08449 $\pm 0.005$	0.13393 $\pm 0.038$	0.06039 $\pm 0.011$	0.18932 $\pm 0.018$

A three-way analysis of variance for these traits (Chalkiness Area (%) and Score) were performed under completely randomized design (CRD) using PROC GLM procedure in SAS. Means were separated using HSD (Tukey’s Studentized Range ) test at p = 0.05. Table includes mean and ± SEM for three way comparison. Chalkiness area (%) was significantly affected by genotype (G) ($p<0.001$), treatment (T) × G ($p<0.001$) and G × panicle type (P) ($p<0.001$) and T × G × P ($p<0.001$) interaction effects. Chalkiness score was significantly affected by G ($p<0.001$), T × G ($p<0.016$), G × P ($p<0.001$) and T × G × P ($p=0.03$) interaction effects

Similar to proportion of chalkiness area, chalkiness score showed an increase under HNT compared to control in IR1561 and WAS-174 in primary and other panicles, while the same was reduced in IR-22 and Kati (Table 10). Among the genotypes, WAS-174 recorded highest percent increase in chalkiness score under HNT in grains from primary (74%) and other panicles (59%) compared to main panicle (Table 10). In contrast, Oryzica recorded an increase in chalkiness score under HNT in grains from primary (46%) and other panicles (99%) compared to main panicle. Genotypes like CO-39, IR1561 and IR-22 showed minimal changes in chalkiness score between tillers under HNT (Table 10). In summary, identifying and using such germplasm (for example, CO-39 and Oryzica) with minimal chalkiness, even under HNT, will help develop rice varieties that can sustain quality under future warming scenarios without having a negative impact of economic revenue of the rice farmers. In addition, the ability to obtain the level of chalkiness in less than 1 s per image and in batch mode allows these models to be used efficiently as a high-throughput phenotyping tool for capturing chalkiness in large breeding populations and to efficiently incorporate genetics leading to low grain chalkiness into ongoing global rice breeding programs.

## Limitations of the study

While the methodology proposed in this study provides tremendous benefits to global rice breeding programs, we would also like to point out its limitations:

- We have shown that the weakly supervised approach, Grad-CAM, can be used to detect chalkiness in white rice (both polished and unpolished grains, with higher accuracy for polished grains, as expected). However, the approach may not work as well for coloured rice such as brown or black rice, given the more opaque nature of coloured rice grains.
- Our experiments showed that models cannot effectively be transferred from polished rice to unpolished rice. Instead, models trained specifically on unpolished rice have better accuracy. This result suggests that new models may need to be trained and fine-tuned (in terms of threshold *T* for binarization and convolution layer to be used for the heatmap) for other types of rice, or for images taken under different conditions.
- The sets of images that were used in this study contained mostly seeds that did not touch each other. The ability of the tool to determine chalkiness in samples without physical separation of grains was not tested in this study. Hence, the tool would require additional training to be able to quantify chalkiness under different proportions of overlap of grains.
- Our approach was designed to determine the global chalkiness in grains, but it does not consider specific chalkiness types such as white-belly, white core, or white-base. However, the developed models set the stage for further refinement to determine the different proportions of chalky types in future work.

## Conclusions

In this study, we presented the application of a high throughput deep learning tool to detect the chalkiness area in polished and unpolished rice grains. To avoid the need for cumbersome pixel-level annotation, we used a weakly supervised segmentation approach, Grad-CAM, which addresses the problem as a binary classification task and subsequently uses the gradients of the grain chalk to produce a chalkiness heatmap. Experimental results showed that it is possible to use the Grad-CAM model with ResNet-101 as a backbone to generate accurate chalkiness heatmaps for both polished and unpolished rice grains. However, the analysis also showed that detecting rice chalkiness is easier in polished rice as compared to unpolished rice and that the polished models are not directly transferable to unpolished rice. Our study shows that weakly supervised deep learning models can be used to assist research in both phenotyping and rice quality control in several ways: (i) perform high-throughput rice seed image analysis to identify chalky seeds and generate chalkiness maps, (ii) replace the expensive error-prone human annotations with rapid and continuous annotations without compromising the accuracy, and (iii) provide quantitative measures for chalkiness area. We successfully demonstrated the application of this tool in accurately capturing the HNT induced differential level of chalkiness in different tillers in rice. The models trained in this study are made publicly available. Being already trained, they will be easy-to-use, scalable and can be readily utilized in ongoing rice breeding programs, without requiring researchers to have computer science or machine learning expertise.

## Supplementary Information

**Additional file 1: Fig. S1.** Steps for rice chalk seed image scanning.

**Additional file 2: Fig. S2.** Image scan of rice seeds.

**Additional file 3: Table S1.** Polished rice seeds statistics.

**Additional file 4: Table S2.** Unpolished rice seeds statistics.

**Additional file 5: Table S3.** Variation of the average IoU with the layer and threshold.

**Additional file 6: Fig. S3.** Examples of Grad-CAM/SqueezeNet-1.0 heatmaps.

**Additional file 7: Fig. S4.** Examples of Grad-CAM/DenseNet-121 heatmaps.

**Additional file 8: Fig. S5.** Examples of Grad-CAM/VGG-19 heatmaps.

**Additional file 9: Fig. S6.**Grad-CAM/Grad-CAM++/Score-CAM heatmaps.

**Additional file 10: Fig. S7.** Examples of predictions on unpolished rice.

**Additional file 11: Fig. S8.** Example of false positive images.

**Additional file 12: Table S4.** Number of grains with and without chalk.

## Data Availability

The datasets generated and analyzed during the current study are available on GitHub, https://github.com/cwang16/Phenotyping-of-Chalkiness-in-Rice.

## Footnotes

1 http://www.knowledgebank.irri.org/ricebreedingcourse/Grain_quality.htm.

2 https://www.amazonaws.cn/en/ec2/instance-types/.

## References

[1] Federation UR. Exporting U.S. Rice. https://www.usarice.com/discover-us-rice/find-a-supplier/exporting-u.s.-rice.

[2] Food supply—crops primary equivalent database. http://www.fao.org/faostat/en/#data/QC.

[3] World Population Prospects 2019: Data Booklet. https://population.un.org/wpp/Publications/Files/WPP2019_DataBooklet.pdf.

[4] The future of food and agriculture. Trends and challenges. http://www.fao.org/3/a-i6583e.pdf

[5] Ray DK, Mueller ND, West PC, Foley JA (2013). Yield trends are insufficient to double global crop production by 2050. PLOS ONE.

[6] Stuecker MF, Tigchelaar M, Kantar MB (2018). Climate variability impacts on rice production in the philippines. PLoS One.

[7] Dabi T, Khanna V (2018). Effect of climate change on rice. Agrotechnology.

[8] Jagadish S, Craufurd P, Wheeler T (2007). High temperature stress and spikelet fertility in rice (*Oryza sativa* L.). J Exp Botany.

[9] Jagadish S, Craufurd P, Wheeler T (2008). Phenotyping parents of mapping populations of rice for heat tolerance during anthesis. Crop Sci.

[10] Jagadish S, Cairns J, Lafitte R, Wheeler TR, Price A, Craufurd PQ (2010). Genetic analysis of heat tolerance at anthesis in rice. Crop Sci.

[11] Bheemanahalli R, Sathishraj R, Tack J, Nalley LL, Muthurajan R, Jagadish KS (2016). Temperature thresholds for spikelet sterility and associated warming impacts for sub-tropical rice. Agric For Meteorol.

[12] Gourdji SM, Sibley AM, Lobell DB (2013). Global crop exposure to critical high temperatures in the reproductive period: historical trends and future projections. Environ Res Lett.

[13] Shi W, Yin X, Struik PC, Solis C, Xie F, Schmidt RC, Huang M, Zou Y, Ye C, Jagadish SVK (2017). High day- and night-time temperatures affect grain growth dynamics in contrasting rice genotypes. J Exp Botany..

[14] Lisle AJ, Martin M, Fitzgerald MA (2000). Chalky and translucent rice grains differ in starch composition and structure and cooking properties. Cereal Chem.

[15] Lyman NB, Jagadish KSV, Nalley LL, Dixon BL, Siebenmorgen T (2013). Neglecting rice milling yield and quality underestimates economic losses from high-temperature stress. PLOS ONE.

[16] Wang K, Li Y, Wang Y, Yang X (2017). On the asymmetry of the urban daily air temperature cycle. J Geophys Res Atmos.

[17] Bahuguna RN, Solis CA, Shi W, Jagadish KS (2017). Post-flowering night respiration and altered sink activity account for high night temperature-induced grain yield and quality loss in rice (*Oryza sativa* l.). Physiologia Plantarum.

[18] Sadok W, Jagadish SK (2020). The hidden costs of nighttime warming on yields. Trends Plant Sci.

[19] Impa SM, Raju B, Hein NT, Sandhu J, Prasad PV, Walia H, Jagadish SK (2021). High night temperature effects on wheat and rice: Current status and way forward.

[20] Lanning SB, Siebenmorgen TJ, Counce PA, Ambardekar AA, Mauromoustakos A (2011). Extreme nighttime air temperatures in 2010 impact rice chalkiness and milling quality. Field Crops Res.

[21] Bahuguna RN, Solis CA, Shi W, Jagadish KSV (2017). Post-flowering night respiration and altered sink activity account for high night temperature-induced grain yield and quality loss in rice (*Oryza sativa* l.). Physiologia Plantarum.

[22] Ashida K, Iida S, Yasui T (2009). Morphological, physical, and chemical properties of grain and flour from chalky rice mutants. Cereal Chem.

[23] Tashiro T, Wardlaw I (1991). The effect of high temperature on kernel dimensions and the type and occurrence of kernel damage in rice. Aust J Agric Res.

[24] Fitzgerald MA, McCouch SR, Hall RD (2009). Not just a grain of rice: the quest for quality. Trends Plant Sci.

[25] Jagadish SVK, Murty MVR, Quick WP (2015). Rice responses to rising temperatures—challenges, perspectives and future directions. Plant Cell Environ.

[26] Bheemanahalli R, Knight M, Quinones C, Doherty CJ, Jagadish SK (2021). Genome-wide association study and gene network analyses reveal potential candidate genes for high night temperature tolerance in rice. Sci Rep.

[27] Su Y, Xiao L. 3d visualization and volume based quantification of rice chalkiness in vivo by using high resolution micro-ct. 2020. 10.21203/rs.2.21396/v1 10.1186/s12284-020-00429-w PMC7494727 32936382

[28] Yang W, Feng H, Zhang X, Zhang J, Doonan JH, Batchelor WD, Xiong L, Yan J (2020). Crop phenomics and high-throughput phenotyping: past decades, current challenges, and future perspectives. Mol Plant.

[29] Komyshev E, Genaev M, Afonnikov D (2017). Evaluation of the seedcounter, a mobile application for grain phenotyping. Front Plant Sci.

[30] Elmasry G, Mandour N, Al-Rejaie S, Belin E, Rousseau D (2019). Recent applications of multispectral imaging in seed phenotyping and quality monitoring—an overview. Sensors.

[31] Walter A, Liebisch F, Hund A (2015). Plant phenotyping: from bean weighing to image analysis. Plant Methods.

[32] Sethy P, Barpanda N, Rath A. Quantification of rice chalkiness using image processing. 2018; 2278–4853.

[33] Alfred R, Lun C. Unsupervised learning of image data using generative adversarial network. In: Joshi X-SYSD (ed.) Advances in Intelligent Systems and Computing. London: Springer. 2019; p. 1041:127–135.

[34] N/A, N.: k-Means advantages and disadvantages—clustering in machine learning. Google. https://developers.google.com/machine-learning/clustering/algorithm/advantages-disadvantages.

[35] Yao Q, Chen J, Guan Z, Sun C, Zhu Z (2009). Inspection of rice appearance quality using machine vision. 2010 Second WRI Glob Congress Intell Syst.

[36] Payman S, Bakhshipour A, Zareiforoush H (2018). Development of an expert vision-based system for inspecting rice quality indices. Qual Assur Safety Crops Foods.

[37] Sun C, Liu T, Ji C, Jiang M, Tian T, Guo D, Wang L, Chen Y, Liang X (2014). Evaluation and analysis the chalkiness of connected rice kernels based on image processing technology and support vector machine. J Cereal Sci.

[38] Chen S, Tao XJ, Guo W, Bu R, Zheng Z, Chen Y, Yang Z, Lin R (2019). Colored rice quality inspection system using machine vision. J Cereal Sci..

[39] Armstrong PR, McClung AM, Maghirang EB, Chen MH, Brabec DL, Yaptenco KF, Famoso AN, Addison CK (2019). Detection of chalk in single kernels of long-grain milled rice using imaging and visible/near-infrared instruments. Cereal Chem.

[40] Jones W, Alasoo K, Fishman D, Parts L (2017). Computational biology: deep learning. Emerg Topics Life Sci.

[41] Tardieu F, Cabrera-Bosquet L, Pridmore T, Bennett M (2017). Plant phenomics, from sensors to knowledge. Curr Biol.

[42] Singh A, Ganapathysubramanian B, Singh A, Sarkar S (2015). Machine learning for high-throughput stress phenotyping in plants. Trends Plant Sci.

[43] He K, Gkioxari G, Dollár P, Girshick R. Mask r-cnn. In: Proceedings of the IEEE International Conference on Computer Vision. 2017; p. 2961–2969.

[44] Xiao J-S, Xu H-H, Ma X-J. Weakly supervised semantic segmentation based on superpixel sampling clustering networks. In: Proceedings of the 2nd International Conference on Computer Science and Software Engineering, Association for Computing Machinery. 2019; p. 178–183.

[45] Selvaraj RR, Cogswell M, Vedantam R, Parikh D, Batra D (2019). Grad-cam: visual explanations from deep networks via gradient-based localization. Int J Comput Vision.

[46] Oquab M, Bottou L, Laptev I, Sivic J. Is object localization for free?-weakly-supervised learning with convolutional neural networks. In: Proceedings of the IEEE Conference on Computer Vision and Pattern Recognition, 2015; p. 685–694.

[47] Wang Y, Zhu F, Boushey CJ, Delp EJ. Weakly supervised food image segmentation using class activation maps. In: 2017 IEEE International Conference on Image Processing (ICIP), New York: IEEE. 2017; p. 1277–1281. 10.1109/ICIP.2017.8296487 PMC6226049 30416395

[48] Li X, Caragea D, Zhang H, Imran M (2019). Localizing and quantifying infrastructure damage using class activation mapping approaches. Soc Netw Anal Mining.

[49] Vinogradova K, Dibrov A, Myers G. Towards interpretable semantic segmentation via gradient-weighted class activation mapping. 2020. arXiv preprint arXiv:2002.11434.

[50] Schumacher M, Genz A, Heinrich M. Weakly supervised pancreas segmentation based on class activation maps. In: Medical Imaging 2020: Image Processing, vol 11313. International Society for Optics and Photonics. 2020; p. 1131314.

[51] Yang S, Kim Y, Kim Y, Kim C. Combinational class activation maps for weakly supervised object localization. In: The IEEE Winter Conference on Applications of Computer Vision, 2020; p. 2941–2949.

[52] Bollis E, Pedrini H, Avila S. Weakly supervised learning guided by activation mapping applied to a novel citrus pest benchmark. In: Proceedings of the IEEE/CVF Conference on Computer Vision and Pattern Recognition Workshops. 2020; p. 70–71

[53] Wang S, Chen W, Xie SM, Azzari G, Lobell DB (2020). Weakly supervised deep learning for segmentation of remote sensing imagery. Remote Sens.

[54] Yang W, Duan L, Chen G, Xiong L, Liu Q (2013). Plant phenomics and high-throughput phenotyping: accelerating rice functional genomics using multidisciplinary technologies. Curr Opin Plant Biol.

[55] United Nation Department of Public Information, U.N.D.: food production must double by 2050 to meet demand from world’s growing population, innovative strategies needed to combat hunger, experts tell second committee. https://www.un.org/press/en/2009/gaef3242.doc.htm

[56] LeCun Y, Boser B, Denker JS, Henderson D, Howard RE, Hubbard W, Jackel LD (1989). Backpropagation applied to handwritten zip code recognition. Neural Comput.

[57] LeCun Y, Bengio Y, Hinton G (2015). Deep learning. Nature.

[58] Goodfellow I, Bengio Y, Courville A, Bengio Y (2016). Deep learning.

[59] Russakovsky O, Deng J, Su H, Krause J, Satheesh S, Ma S, Huang Z, Karpathy A, Khosla A, Bernstein M, Berg AC, Fei-Fei L (2015). ImageNet large scale visual recognition challenge. Int J Comput Vis (IJCV).

[60] Krizhevsky A, Sutskever I, Hinton G (2012). Imagenet classification with deep convolutional neural networks. Neural Inform Process Syst.

[61] Simonyan K, Zisserman A. Very deep convolutional networks for large-scale image recognition. 2014. arXiv:1409.1556.

[62] He K, Zhang X, Ren S, Sun J. Deep residual learning for image recognition. 2016; p. 770–778. 10.1109/CVPR.2016.90.

[63] Iandola F, Han S, Moskewicz M, Ashraf K, Dally W, Keutzer K. Squeezenet: Alexnet-level accuracy with 50x fewer parameters and < 0.5mb model size. 2016.

[64] Huang G, Liu Z, van der Maaten L, Weinberger K. Densely connected convolutional networks. 2017. 10.1109/CVPR.2017.243.

[65] Tan M, Le Q. Efficientnet: rethinking model scaling for convolutional neural networks. 2019.

[66] Selvaraju RR, Cogswell M, Das A, Vedantam R, Parikh D, Batra D (2019). Grad-cam: visual explanations from deep networks via gradient-based localization. Int J Comput Vision.

[67] Zhou B, Khosla A, Lapedriza A, Oliva A, Torralba A. Learning deep features for discriminative localization. In: Proceedings of the IEEE Conference on Computer Vision and Pattern Recognition. 2016; p. 2921–2929.

[68] Zhou B, Khosla A, Lapedriza A, Oliva A, Torralba A. Learning deep features for discriminative localization. 2015. arXiv:1512.04150.

[69] Singh KK, Lee YJ. Hide-and-seek: forcing a network to be meticulous for weakly-supervised object and action localization. In: 2017 IEEE International Conference on Computer Vision (ICCV), New York: IEEE. 2017; p. 3544–3553.

[70] Zhang X, Wei Y, Kang G, Yang Y, Huang T. Self-produced guidance for weakly-supervised object localization. In: Proceedings of the European Conference on Computer Vision (ECCV). 2018; p. 597–613.

[71] Zhang X, Wei Y, Feng J, Yang Y, Huang TS. Adversarial complementary learning for weakly supervised object localization. In: Proceedings of the IEEE Conference on Computer Vision and Pattern Recognition. 2018; p. 1325–1334.

[72] Choe J, Shim H. Attention-based dropout layer for weakly supervised object localization. In: Proceedings of the IEEE/CVF Conference on Computer Vision and Pattern Recognition. 2019; pp. 2219–2228.

[73] Zhang Y, Hong D, McClement D, Oladosu O, Pridham G, Slaney G (2021). Grad-cam helps interpret the deep learning models trained to classify multiple sclerosis types using clinical brain magnetic resonance imaging. J Neurosci Methods.

[74] Ruiz I, Porzi L, Bulò SR, Kontschieder P, Serrat J. Weakly supervised multi-object tracking and segmentation. In: WACV (Workshops). 2021; p. 125–133.

[75] Nunnari F, Kadir MA, Sonntag D. On the overlap between grad-cam saliency maps and explainable visual features in skin cancer images. In: International Cross-Domain Conference for Machine Learning and Knowledge Extraction. Springer: Cham. 2021; p. 241–253.

[76] Daanouni O, Cherradi B, Tmiri A. Automatic detection of diabetic retinopathy using custom cnn and grad-cam. In: Advances on smart and soft computing. Springer: Cham. 2021; p. 15–26.

[77] Joshua ESN, Chakkravarthy M, Bhattacharyya D. Lung cancer detection using improvised grad-cam++ with 3d cnn class activation. In: Smart technologies in data science and communication. Springer: Cham, 2021; p. 55–69.

[78] Wang H, Wang Z, Du M, Yang F, Zhang Z, Ding S, Mardziel P, Hu X. Score-cam: score-weighted visual explanations for convolutional neural networks. In: Proceedings of the IEEE/CVF Conference on Computer Vision and Pattern Recognition Workshops. 2020. pp. 24–25.

[79] Ren S, He K, Girshick RB, Sun J. Faster r-cnn: towards real-time object detection with region proposal networks. In: NIPS, 2015. 10.1109/TPAMI.2016.2577031 27295650

[80] Minaee S, Boykov YY, Porikli F, Plaza AJ, Kehtarnavaz N, Terzopoulos D. Image segmentation using deep learning: a survey. IEEE transactions on pattern analysis and machine intelligence; 2021. 10.1109/TPAMI.2021.3059968 33596172

[81] Pillay N, Gerber M, Holan K, Whitham SA, Berger DK. Quantifying the severity of common rust in maize using mask r-cnn. In: International Conference on Artificial Intelligence and Soft Computing. Springer: Cham. 2021; p. 202–213.

[82] Bheemanahalli R, Wang C, Bashir E, Chiluwal A, Pokharel M, Perumal R, Moghimi N, Ostmeyer T, Caragea D, Jagadish S. Classical phenotyping and deep learning concur on genetic control of stomatal density and area in sorghum. Plant Physiol. 2021. 10.1093/plphys/kiab174 PMC8260133 33856488

[83] Kundu A, Mishra C, Bilgaiyan S. Covid-segnet: Diagnosis of covid-19 cases on radiological images using mask r-cnn. In: 2021 Seventh International Conference on Bio Signals, Images, and Instrumentation (ICBSII), New York: IEEE. 2021; p. 1–5.

[84] Albuquerque CK, Polimante S, Torre-Neto A, Prati RC. Water spray detection for smart irrigation systems with mask r-cnn and uav footage. In: 2020 IEEE International Workshop on Metrology for Agriculture and Forestry (MetroAgriFor). New York: IEEE; 2020. p. 236–240.

[85] Lv Y, Zhang C, Yun W, Gao L, Wang H, Ma J, Li H, Zhu D (2020). The delineation and grading of actual crop production units in modern smallholder areas using rs data and mask r-cnn. Remote Sens.

[86] Šebela D, Bheemanahalli R, Tamilselvan A, Kadam NN, Jagadish SK (2019). Genetic dissection of photochemical efficiency under water-deficit stress in rice. Plant Physiol Rep.

[87] He K, Gkioxari G, Dollar P, Girshick R. Mask r-cnn. IEEE Transactions on Pattern Analysis and Machine Intelligence. 2018, p. 1–1. 10.1109/TPAMI.2018.2844175. 10.1109/TPAMI.2018.2844175 29994331

[88] Dutta A, Zisserman A. The via annotation software for images, audio and video. 2019; p. 2276–2279 . 10.1145/3343031.3350535.

[89] Shelhamer E, Long J, Darrell T (2016). Fully convolutional networks for semantic segmentation. IEEE Trans Pattern Anal Mach Intell.

[90] Russakovsky O, Deng J, Su H, Krause J, Satheesh S, Ma S, Huang Z, Karpathy A, Khosla A, Bernstein M (2015). Imagenet large scale visual recognition challenge. Int J Comput Vision.

[91] Souibgui MA, Kessentini Y. De-gan: a conditional generative adversarial network for document enhancement. IEEE Transactions on Pattern Analysis and Machine Intelligence. 2020. 10.1109/TPAMI.2020.3022406 32894707

[92] Alijla BO, Saad M, Issawi SF. Neural network-based minutiae extraction for fingerprint verification system. In: 2017 8th International Conference on Information Technology (ICIT). New York: IEEE; 2017. p. 435–441.

[93] Huang M-L, Fu C-C (2018). Applying image processing to the textile grading of fleece based on pilling assessment. Fibers.

[94] Shi W, Muthurajan R, Rahman H, Selvam J, Peng S, Zou Y, Jagadish KS (2013). Source-sink dynamics and proteomic reprogramming under elevated night temperature and their impact on rice yield and grain quality. N Phytologist.

