# Supplementary material for: Deep learning based high-throughput phenotyping of chalkiness in rice exposed to high night temperature
Source: Plant Methods. 2022 Jan 22;18:9. doi: 10.1186/s13007-022-00839-5 (PMC8783510; doi:10.1186/s13007-022-00839-5)
Supplement: Supplementary file 1 — Additional file 1: Fig. S1. Steps for rice chalk seed image scanning. [file 13007_2022_839_MOESM1_ESM.pdf]

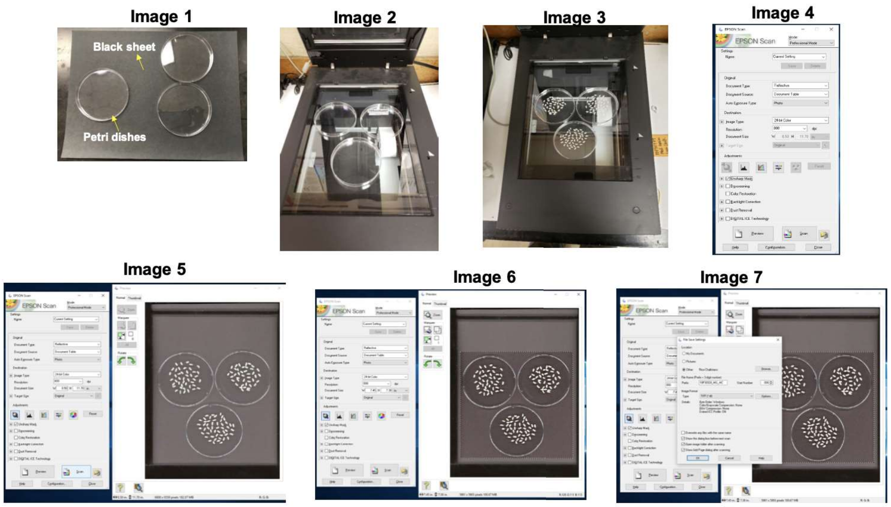

Figure S1 Steps for rice chalk seed image scanning using Epson Perfection V800 photo scanner.

Image 1: Showing the materials required: Rice seeds (dehusked), Epson perfection V800 photo scanner attached with the computer, Petri-dishes, non-metal forceps, a black sheet of paper (A4).

Steps for image scanning of both polished and unpolished grains

- Place the transparent Petri-dishes on the scanner away from the edges of the scanner, but not touching each other **(See image 2)**
- Spread the rice seeds in the middle of the Petri-dishes **(See image 3)**
- Place the black sheet of paper over dishes and shut the scanner lid
- Open the computer and scanner
- Click the scanner software desktop icon

Scanner setting (See image 4)

- Start Epson Scan and select Professional Mode as the Mode setting
- Select these settings under the Original section:
  - Document Type setting = "Reflective"
  - Select the Document source = "Document Table"
  - Select Auto Exposure type = "Photo."
- Select these settings in the Destination section:
  - Select the image type = "24-bit Color"

  - Set resolution dpi as - 800 and click OK (do not change the dpi, keep this as constant across scans)
- Select these settings in the Adjustments section:
  - Check Unsharp Mask
  - Uncheck all other options.
- Click 'PREVIEW' to see the scan
- If the dishes are all within the scan area and all of the rice grains are away from the edges of the dishes, then continue to the next step. If not, readjust the location of dishes and/or rice and click preview again. (**See image 5**)
- Using the mouse, left click and drag to create a box around the Petri-dishes in the scan preview. This will set the scanning area for the final scan. Should include all borders of the Petri-dishes but not excessive blank area outside of the dishes. (**See image 6**)
- Return to the Main window and Click 'SCAN'.
- In the Location, setting, click on "Other" and then Browse... to select folder location the images should be saved. (**See image 7**)
- In the File Name section type file name in Prefix and set start number to 1. Start Number will automatically increase with each scan.
- In the Image Format Section, choose file type TIFF (*.tif)
- In the bottom section:
  - Uncheck "Overwrite any files with the same name."
  - Check "Show this dialog box before next scan."
  - Check "Open image folder after scanning"
  - Check "Show Add Page dialog after scanning"
- Click OK.
- After scan, return rice back to packets and begin again at the top of the page until the number of replications has been reached, or begin on next sample.
