# Supplementary material for: Deep learning based high-throughput phenotyping of chalkiness in rice exposed to high night temperature
Source: Plant Methods. 2022 Jan 22;18:9. doi: 10.1186/s13007-022-00839-5 (PMC8783510; doi:10.1186/s13007-022-00839-5)
Supplement: Supplementary file 2 — Additional file 2: Fig. S2. Image scan of rice seeds. [file 13007_2022_839_MOESM2_ESM.pdf]

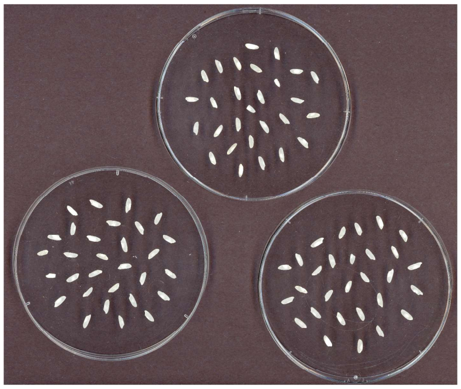

Figure S2 Image scan of rice seeds spread on three Petri dishes covered with a black background. The seeds on the three dishes correspond to one size/chalkiness combination for polished rice, and one genotype/tiller/condition for unpolished rice, respectively. Three such images were obtained for each combination resulting in three replicates, each with different seeds.
