# Supplementary material for: Deep learning based high-throughput phenotyping of chalkiness in rice exposed to high night temperature
Source: Plant Methods. 2022 Jan 22;18:9. doi: 10.1186/s13007-022-00839-5 (PMC8783510; doi:10.1186/s13007-022-00839-5)
Supplement: Supplementary file 3 — Additional file 3: Table S1. Polished rice seeds statistics. [file 13007_2022_839_MOESM3_ESM.pdf]

Table S1 Polished rice grains statistics. For each combination of grain size (column 1), chalkiness degree (column 2) and replicate (column 3), the total number of grains in the corresponding high resolution image and the number of grains used in the analysis are shown in columns 4 and 5, respectively. Columns 6 and 7 show the number of (used) grains annotated as chalky and non-chalky, respectively.

Grain size	Chalkiness degree	Replicate	Grains original	Grains used	Chalky	Non-chalky
long	low	1	76	76	27	49
long	low	2	96	96	27	69
long	low	3	88	88	25	63
long	medium	1	96	96	21	75
long	medium	2	102	102	31	71
long	medium	3	85	85	23	62
long	high	1	87	81	61	20
long	high	2	91	91	73	18
long	high	3	88	88	65	23
medium	low	1	90	90	21	69
medium	low	2	97	97	17	80
medium	low	3	80	80	17	63
medium	medium	1	106	106	25	81
medium	medium	2	105	105	33	72
medium	medium	3	99	99	24	75
medium	high	1	66	66	46	20
medium	high	2	100	100	61	39
medium	high	3	102	99	63	36
		Total	1654	1645	660	985
