# Supplementary material for: Deep learning based high-throughput phenotyping of chalkiness in rice exposed to high night temperature
Source: Plant Methods. 2022 Jan 22;18:9. doi: 10.1186/s13007-022-00839-5 (PMC8783510; doi:10.1186/s13007-022-00839-5)
Supplement: Supplementary file 4 — Additional file 4: Table S2. Unpolished rice seeds statistics. [file 13007_2022_839_MOESM4_ESM.pdf]

Table S2 Unpolished rice grains statistics. For each combination of genotype (column 1), tiller (column 2), condition (column 3) and replicate (column 4), the total number of grains in the corresponding high resolution image and the number of grains used in the analysis are shown in columns 5 and 6, respectively. Columns 7 and 8 show the number of (used) grains annotated as chalky and non-chalky, respectively.

Genotype	Tiller	Treatment	Replicate	Grains original	Grains used	Chalky	Non-chalky
Kati	Main	CNT	1	139	138	122	16
		CNT	2	160	160	101	59
		CNT	3	95	95	63	32
		HNT	1	169	165	158	7
		HNT	2	161	160	139	21
		HNT	3	152	147	119	28
	Primary	CNT	1	152	152	121	31
		CNT	2	137	137	92	45
		CNT	3	150	150	117	33
		HNT	1	148	148	145	3
		HNT	2	146	146	122	24
		HNT	3	149	149	136	13
	Other	CNT	1	133	133	115	18
		CNT	2	136	136	88	48
		CNT	3	132	132	79	53
		HNT	1	154	152	145	7
		HNT	2	166	166	132	34
		HNT	3	148	148	138	10
CO-39	Main	CNT	1	166	166	104	62
		CNT	2	86	86	5	81
		CNT	3	125	123	98	25
		HNT	1	164	164	155	9
		HNT	2	89	89	13	76
		HNT	3	186	186	130	56
	Primary	CNT	1	167	167	128	39
		CNT	2	150	148	48	100
		CNT	3	145	145	106	39
		HNT	1	148	148	138	10
		HNT	2	149	148	99	49
		HNT	3	155	150	118	32
	Other	CNT	1	150	150	134	16
		CNT	2	152	151	117	34
		CNT	3	154	154	110	44
		HNT	1	152	152	130	22
		HNT	2	147	147	55	92
		HNT	3	145	145	114	31

Table T2 Continued

Genotype	Tiller	Treatment	Replicate	Grains original	Grains used	Chalky	Non-chalky
IR-22	Main	CNT	1	97	97	0	97
		CNT	2	86	86	0	86
		HNT	1	97	96	0	96
		HNT	2	50	50	0	50
		HNT	3	43	43	0	43
	Primary	CNT	1	133	133	1	132
		CNT	2	156	155	0	155
		HNT	1	152	152	1	151
		HNT	2	90	90	0	90
		HNT	3	95	95	1	94
	Other	CNT	1	155	155	0	155
		CNT	2	150	150	0	150
		CNT	3	105	104	0	104
		HNT	1	152	152	1	151
		HNT	2	152	150	0	150
		HNT	3	148	148	0	148
IR-1561	Main	CNT	1	124	124	0	124
		CNT	2	97	97	0	97
		CNT	3	116	116	1	115
		HNT	1	92	92	1	91
		HNT	2	85	85	0	85
		HNT	3	26	26	1	25
	Primary	CNT	1	152	151	0	151
		CNT	2	148	148	0	148
		CNT	3	149	149	0	149
		HNT	1	104	103	3	100
		HNT	2	161	157	1	156
		HNT	3	21	21	0	21
	Other	CNT	1	151	151	0	151
		CNT	2	143	142	0	142
		CNT	3	148	148	0	148
		HNT	1	160	160	24	136
		HNT	2	151	151	1	150
		HNT	3	146	146	1	145

Table T2 Continued

Genotype	Tiller	Treatment	Replicate	Grains original	Grains used	Chalky	Non-chalky
WAS-174	Main	CNT	1	116	115	6	109
		CNT	2	180	180	0	180
		CNT	3	195	194	0	194
		HNT	1	127	127	6	121
		HNT	2	120	120	3	117
		HNT	3	155	155	0	155
	Primary	CNT	1	145	145	6	139
		CNT	2	159	154	0	154
		CNT	3	152	150	0	150
		HNT	1	156	156	4	152
		HNT	2	152	149	15	134
		HNT	3	157	157	2	155
	Other	CNT	1	157	157	4	153
		CNT	2	150	150	0	150
		CNT	3	141	141	1	140
		HNT	1	155	155	9	146
		HNT	2	141	141	3	138
		HNT	3	153	153	0	153
Oryzica	Main	CNT	1	54	54	3	51
		CNT	2	40	40	0	40
		CNT	3	55	55	0	55
		HNT	1	25	25	6	19
		HNT	2	52	52	0	52
		HNT	3	64	64	0	64
	Primary	CNT	1	77	77	3	74
		CNT	2	59	59	1	58
		CNT	3	71	71	0	71
		HNT	1	32	32	12	20
		HNT	2	100	100	2	98
		HNT	3	89	89	1	88
	Other	CNT	1	141	141	8	133
		CNT	2	89	89	0	89
		CNT	3	68	68	0	68
		HNT	1	34	34	15	19
		HNT	2	41	41	2	39
		HNT	3	55	55	2	53
		Total		13149	13101	4085	9016
