# Supplementary material for: Deep learning based high-throughput phenotyping of chalkiness in rice exposed to high night temperature
Source: Plant Methods. 2022 Jan 22;18:9. doi: 10.1186/s13007-022-00839-5 (PMC8783510; doi:10.1186/s13007-022-00839-5)
Supplement: Supplementary file 5 — Additional file 5: Table S3. Variation of the average IoU with the layer and threshold. [file 13007_2022_839_MOESM5_ESM.pdf]

Table S3 Variation of the Average IoU (%) with the layer and threshold used for DenseNet-121, SqueezeNet-1.0, VGG-16 and EfficientNetB4, respectively. The layer is used to generate the heatmaps and the threshold T is used to binarize the heatmaps (e.g., $T = 20\%$ means that only pixels with values at least 20% of the max pixel value in the image are included in the binary mask). The layers were sampled to include a low-level layer, a high-level layer and two intermediate layers that showed good results based on a qualitative inspection of the maps. The threshold T is varied from 20% to 80% in increments of 10. The best result and the corresponding layer and threshold for each network are highlighted in bold blue font.

DenseNet-121							
Layer	T=20%	T=30%	T=40%	T=50%	**T=60%**	T=70%	T=80%
denseblock1_denselayer6_conv2	0.00%	0.54	5.51	11.94	36.48	18.23	18.23
**denseblock2_denselayer7_conv2**	0.00	28.04	11.82	25.52	**47.44**	18.55	18.55
denseblock3_denselayer7_conv2	0.09	8.40	16.55	26.38	42.33	18.23	18.23
features_transition1_conv	0.00	2.80	12.94	25.58	40.34	18.23	18.23
SqueezeNet-1.0							
Layer	T=20%	T=30%	T=40%	T=50%	**T=60%**	T=70%	T=80%
features_3_expand1x1	0.42	0.63	1.46	6.96	26.28	18.55	18.55
features_4_expand1x1	0.00	2.73	9.35	22.26	20.25	18.55	18.55
features_8_expand1x1	0.00	2.61	3.57	5.92	23.41	18.55	18.55
**features_12_expand1x1**	0.03	4.62	10.00	18.14	**31.01**	18.55	18.55
VGG-16							
Layer	T=20%	T=30%	T=40%	T=50%	**T=60%**	T=70%	T=80%
**features_module_5**	0.16	8.63	13.69	18.57	**24.92**	18.55	18.55
features_module_7	0.00	0.00	0.06	1.82	18.32	18.55	18.55
features_module_14	0.00	0.00	0.04	2.78	17.05	18.55	18.55
features_module_21	0.00	0.01	0.03	1.95	14.59	18.55	18.55
EfficientNetB4							
Layer	T=20%	T=30%	T=40%	**T=50%**	T=60%	T=70%	T=80%
block1a_se_excite	0.00	0.05	1.03	18.55	18.55	18.55	18.55
block2a_expand_conv	0.00	0.00	1.38	30.44	30.44	18.55	18.55
block2b_expand_conv	0.00	0.02	0.48	17.11	17.65	18.55	18.55
**stem_conv**	0.00	0.00	0.04	**35.40**	28.09	18.55	18.55
