# Supplementary material for: Deep learning based high-throughput phenotyping of chalkiness in rice exposed to high night temperature
Source: Plant Methods. 2022 Jan 22;18:9. doi: 10.1186/s13007-022-00839-5 (PMC8783510; doi:10.1186/s13007-022-00839-5)
Supplement: Supplementary file 6 — Additional file 6: Fig. S3. Examples of Grad-CAM/SqueezeNet-1.0 heatmaps. [file 13007_2022_839_MOESM6_ESM.pdf]

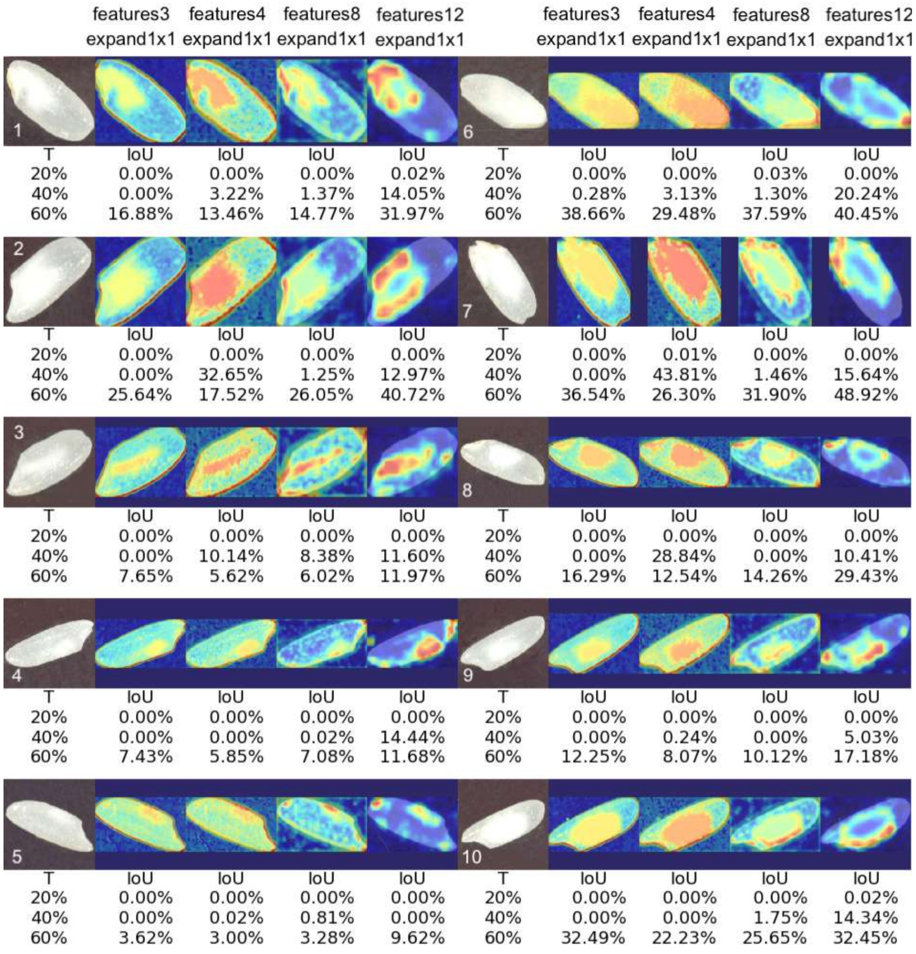

Figure S3 Examples of Grad-CAM (SqueezeNet-1.0) heatmaps for 10 sample chalky seed images (5 on the left side and 5 on the right side). For each seed, heatmaps corresponding to the following four layers are shown: (1) squeezenet1_0 features_3_expand1x1; (2) squeezenet1_0 features_4_expand1x1; (3) squeezenet1_0 features_8_expand1x1; (4) squeezenet1_0 features_12_expand1x1. The IoU values obtained for three thresholds T (20%, 40% and 60%, respectively) are shown under each heatmap.
