# Supplementary figures and images for: Deep learning based high-throughput phenotyping of chalkiness in rice exposed to high night temperature
Source: Plant Methods. 2022 Jan 22;18:9. doi: 10.1186/s13007-022-00839-5 (PMC8783510; doi:10.1186/s13007-022-00839-5)

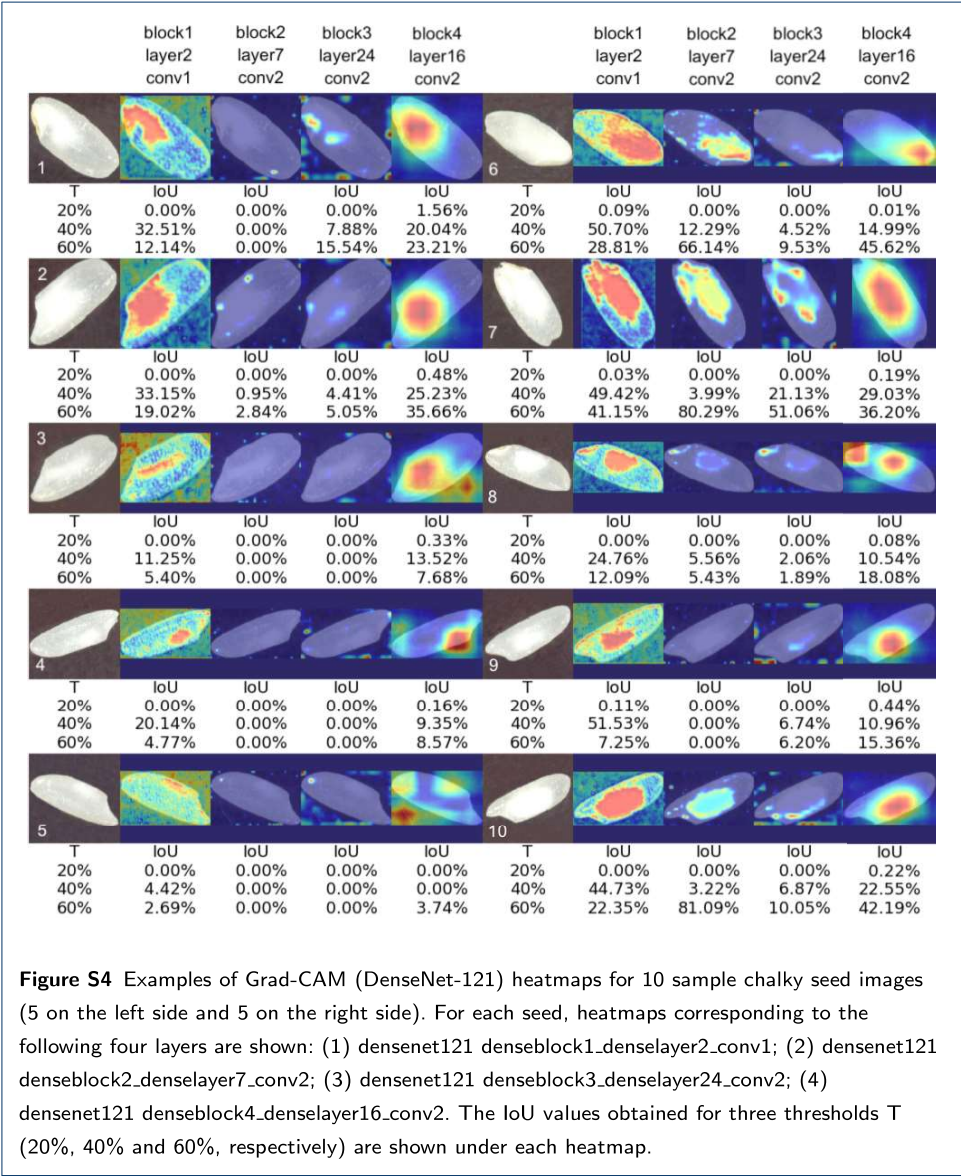

Supplement: Supplementary file 7 — Additional file 7: Fig. S4. Examples of Grad-CAM/DenseNet-121 heatmaps. [file 13007_2022_839_MOESM7_ESM.pdf]

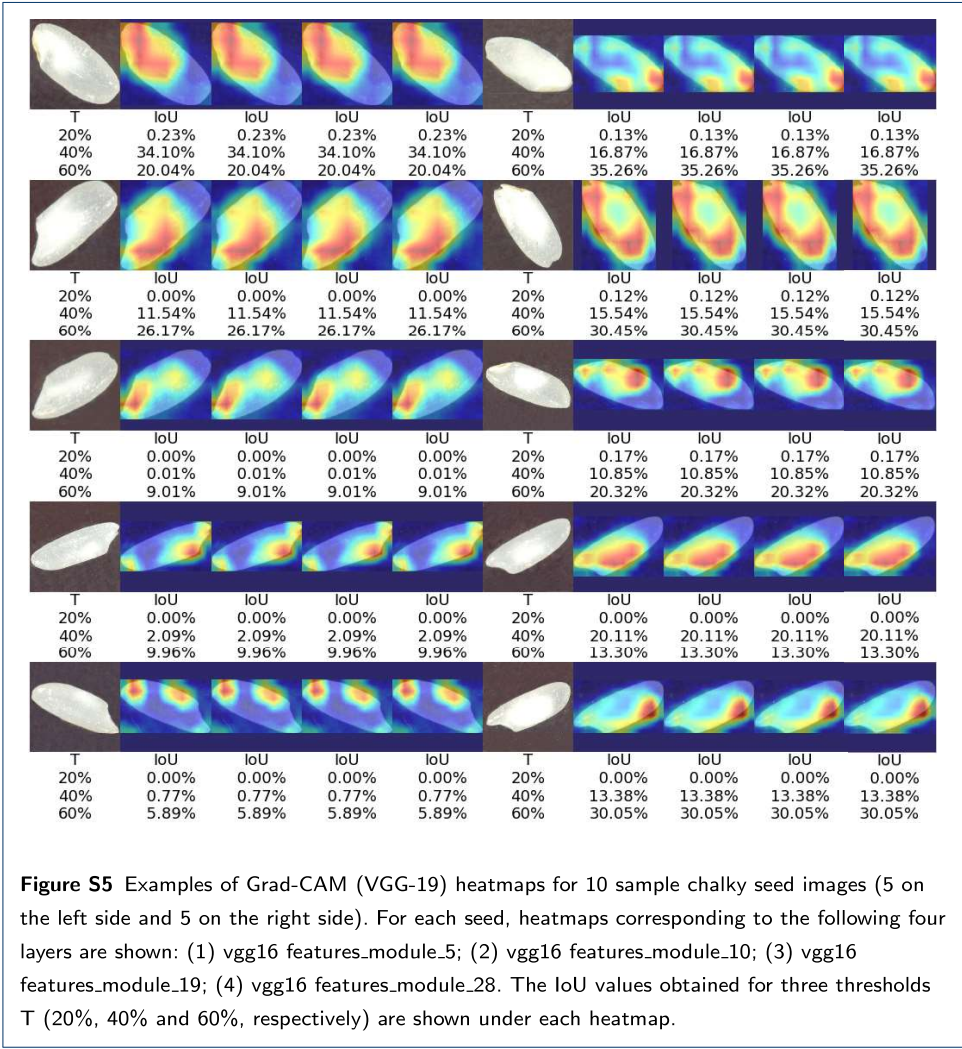

Supplement: Supplementary file 8 — Additional file 8: Fig. S5. Examples of Grad-CAM/VGG-19 heatmaps. [file 13007_2022_839_MOESM8_ESM.pdf]

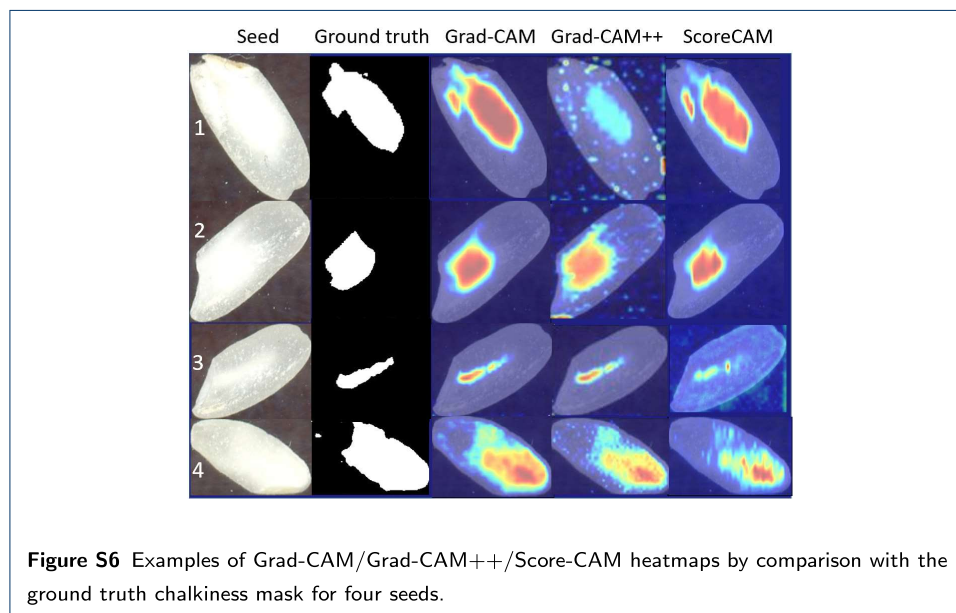

Supplement: Supplementary file 9 — Additional file 9: Fig. S6.Grad-CAM/Grad-CAM++/Score-CAM heatmaps. [file 13007_2022_839_MOESM9_ESM.pdf]
