# Supplementary material for: Deep learning based high-throughput phenotyping of chalkiness in rice exposed to high night temperature
Source: Plant Methods. 2022 Jan 22;18:9. doi: 10.1186/s13007-022-00839-5 (PMC8783510; doi:10.1186/s13007-022-00839-5)
Supplement: Supplementary file 10 — Additional file 10: Fig. S7. Examples of predictions on unpolished rice. [file 13007_2022_839_MOESM10_ESM.pdf]

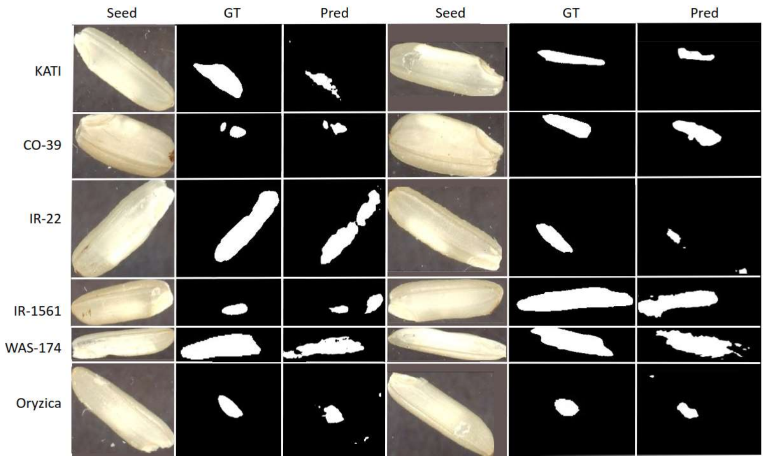

Figure S7 Examples of binary masks predicted by Grad-CAM on 12 unpolished images, by comparison with the ground truth binary masks. The Grad-CAM model used was trained on the 12 combinations corresponding to CO-39 and Kati genotypes. The images shown are randomly selected from the other four genotypes not included in the training. These examples show that the unpolished model generalizes well from some genotypes to others.
