# Supplementary material for: Deep learning based high-throughput phenotyping of chalkiness in rice exposed to high night temperature
Source: Plant Methods. 2022 Jan 22;18:9. doi: 10.1186/s13007-022-00839-5 (PMC8783510; doi:10.1186/s13007-022-00839-5)
Supplement: Supplementary file 11 — Additional file 11: Fig. S8. Example of false positive images. [file 13007_2022_839_MOESM11_ESM.pdf]

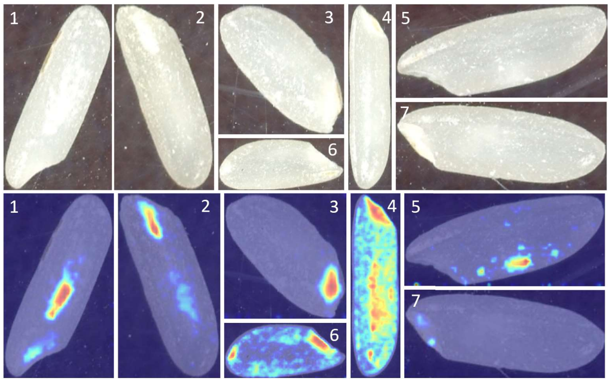

Figure S8 Examples of false positive images, i.e., non-chalky seed images that are predicted as chalky by ResNet-101. For each seed, the corresponding chalkiness heatmap identified by Grad-CAM is also shown. The misclassification is generally due to what the experts who annotated the images manually considered to be abrasion (e.g., images 1, 2, 4, 5), or damaged tips/ends (e.g., images 3, 6, 7).
