# Supplementary material for: Deep learning based high-throughput phenotyping of chalkiness in rice exposed to high night temperature
Source: Plant Methods. 2022 Jan 22;18:9. doi: 10.1186/s13007-022-00839-5 (PMC8783510; doi:10.1186/s13007-022-00839-5)
Supplement: Supplementary file 12 — Additional file 12: Table S4. Number of grains with and without chalk. [file 13007_2022_839_MOESM12_ESM.pdf]

	% Chalkiness	
**Genotype**	**Control**	**HNT**
**CO-39**	63.2 ± 9	68.8 ± 9
**IR-1561**	0.1 ± 0.1	2.76 ± 1.6
**IR-22**	0.1 ± 0.09	0.26 ± 0.14
**Kati**	72.6 ± 3.5	89.4 ± 2.3
**Oryzica**	1.87 ± 0.83	13.0 ± 5.8
**WAS-174**	1.4 ± 0.7	3.2 ± 1
**Treatment (T)**	0.005	
**Genotype (G)**	$<$0.001	
**T * G**	0.2391	

Table S4 Number of seeds with and without chalk selected across treatments and genotypes. A three-way analysis of variance for this trait was performed under completely randomized design (CRD) using PROC GLM procedure in SAS. Means were separated using HSD (Tukey's Studentized Range) test at p=0.05. Table includes mean and +/- SEM for three way comparison. No significant differences were found.
